# Radiofrequency EMF irradiation effects on pre-B lymphocytes undergoing somatic recombination

**DOI:** 10.1038/s41598-021-91790-3

**Published:** 2021-06-16

**Authors:** Elena Ioniţă, Aurelian Marcu, Mihaela Temelie, Diana Savu, Mihai Şerbănescu, Mihai Ciubotaru

**Affiliations:** 1 Department of Physics of Life and Environmental Sciences, Horia Hulubei National Institute for R&D in Physics and Nuclear Engineering, 077125 Măgurele, Ilfov Romania; 2 Department of Immunology, Internal Medicine, Colentina Clinical Hospital, 72202 Bucharest, Romania; 3 Center for Advanced Laser Technologies, National Institute for Laser Plasma and Radiation Physics, 077125 Măgurele, Ilfov Romania

**Keywords:** Biophysics, Immunology, Molecular biology, Risk factors

## Abstract

Intense electromagnetic fields (EMFs) induce DNA double stranded breaks (DSBs) in exposed lymphocytes.We study developing pre-B lymphocytes following V(D)J recombination at their Immunoglobulin light chain loci (*IgL*). Recombination physiologically induces DNA DSBs, and we tested if low doses of EMF irradiation affect this developmental stage. Recombining pre-B cells, were exposed for 48 h to low intensity EMFs (maximal radiative power density flux S of 9.5 µW/cm^2^ and electric field intensity 3 V/m) from waves of frequencies ranging from 720 to 1224 MHz. Irradiated pre-B cells show decreased levels of recombination, reduction which is dependent upon the power dose and most remarkably upon the frequency of the applied EMF. Although 50% recombination reduction cannot be obtained even for an S of 9.5 µW/cm^2^ in cells irradiated at 720 MHz, such an effect is reached in cells exposed to only 0.45 µW/cm^2^ power with 950 and 1000 MHz waves. A maximal four-fold recombination reduction was measured in cells exposed to 1000 MHz waves with S from 0.2 to 4.5 µW/cm^2^ displaying normal levels of γH2AX phosphorylated histone. Our findings show that developing B cells exposure to low intensity EMFs can affect the levels of production and diversity of their antibodies repertoire.

## Introduction

Somatic or V(D)J recombination is the process that assembles in all jawed vertebrates the gene segments encoding the variable regions of the specific antigen immune receptors (T cell and Immunoglobulin IG) of the lymphoid T and B cells^1^. This process occurs in lymphocyte precursors, is mediated by RAG (recombination activating gene proteins) recombinase a heterotetrameric complex made of a dimer of RAG1 and two monomers of RAG2^2,3^. RAG1 a member of the DDE transposase/Integrase family is the key catalytic component of RAG. RAG binds specifically to recombination signal sequences (RSS) flanking germinal coding V, (D), J gene segments in the variable region at the IG and T cell receptor loci and catalyzes their rearrangement^4^. RAG recombination generates two DNA hairpins at the coding ends and two blunt double stranded DNA cuts at the signal ends. RAG maintains the paired cleaved ends in proximity and allows the ubiquitous set of non-homologous end-joining (NHEJ) DNA repair enzymes (Artemis, ATM, DNAPk, XRCC4, DNA Ligase IV) to resolve the hairpins and join the cleaved ends. For B and T lymphocytes recombination occurs at two stages during their differentiation^5^. We will discuss only the B lineage development in the bone marrow. First two rounds, D to J (in pre-pro stage) followed by V to DJ recombination (in late-pro stage) occur in pro-B cells at their Ig Heavy chain locus (*IgH*). Once *IgH* locus is rearranged, expressed Igµ together with a surrogate light chain comprising λ5 Vpre B proteins and two Igα, β signaling subunits assemble the pre-B cell receptor(pre-BCR)^6^, which marks the large pre-B cell stage. Stromal bone marrow cells secreted interleukin IL-7 binds to their receptor (IL-7R), a signal which is transduced as pro-survival and proliferative^7^. First, IL-7R signals through Janus Kinase 3-(JAK-3)^8^ phosphorylating and recruiting the signal transducer and activator of transcription 5A and B (STAT5A and B)^9,10^ which stimulate transcription of *Ccnd3* encoding Cyclin D3^11^ and of the B cell lymphoma 2(bcl2) gene^12^. Both Cyclin D3 and the anti-apoptotic BCL2 help pre-B cells through cell cycle G1 checkpoint allowing the replication of their DNA. Secondly, IL-7R signals in large pre-B cells through phosphoinositide 3-kinase (PI3K)^13^ and protein Kinase B (AKT) phosphorylating the forkhead box O 1, 3 (FOXO1,3) transcription factors, modification which exports them from nuclei and targets the proteins for degradation^14–16^. FOXO1, 3 activate *e-rag* enhancer and *rag1*, *2* genes transcription^14,17^. In large pre-B cells IL-7R also signals via the nuclear factor kappa light chain enhancer of activated B cells (NF-kB) stimulated by AKT phosphorylation of IKKα serine 23^18^. NF-kB activates Cyclin D4 kinase which targets FOXO1 for phosphorylation and repression^19^. By inhibiting FOXO1, or phosphorylating STAT-5, IL-7R signals are transiently downregulating RAG proteins in large pre-B cells. After four to five rounds of replication the large pre-B lymphocytes get under the influence of cell surface pre-BCR receptor aggregation and stimulation (in absence of a bonified ligand), a signal which antagonizes that of IL-7R, induces cell cycle arrest and transitions cells towards small pre-B stage^20^. Stimulation of pre-BCR cascades through RAS and extracellular signal- regulated kinase (ERK) upregulating the E2A transcription factor expression. E2A binds both Igk intronic and Igk 3’ enhancers making the *Igk* light chain locus accessible for recombination^21^. Another effect of pre-BCR stimulation signals through spleen tyrosine kinase(SYK) and B cell-linker protein(BLNK) which together repress PI3K and AKT but stimulate mitogen activated p38 kinase which activates FOXO1 to express RAG^13,20,22^. Consequently, in small pre-B cells subsequent V to J rearrangements occur at *Ig L k* or *λ* light chain loci. Upon completion of a successful V to J recombined allele, the cell develops into naïve immature B cell, exposing IgM B cell receptors (BCR).

Interference of V(D)J recombination with other concurrent exogenous factors favoring DNA DSBs, like ionizing or EM irradiation can induce DNA damage which may lead to oncogenic translocations such as those described in acute lymphoblastic leukemia (ALL)^23,24^. Exposure of human blood lymphocytes from healthy donors to strong EMFs (2 h irradiation with sinusoidal pulses at 4 × 10^5^ V/m 50 Hz with a carrier wave of 10 Hz^25^) causes DNA DSBs and chromosomal lesions whose severity correlate with the intensity of the applied fields and the duration of exposure. However, less clear results come from studies with irradiated lymphocytes using low intensity, high radiofrequency(RF) EMFs (3 kHz–300 GHz)^26^. Most of these studies have assessed the levels of EMF inflicted DNA single and DSBs on lymphocytes using the microgel electrophoresis technique or ‘comet assay’, which detects brakes with a sensitivity limit of 50 strand events per diploid cell^27^. RF EM irradiation from cell phones was first studied by Phillips et al. in Molt-4 human lymphoblastoid cells exposed for 2–21 h to fields of 813.5 and 836.5 MHz with specific absorption rate (SAR) (2.4–26 µW/g)^28^. Variable degree of DNA damage is reported, mainly induced by high SAR values waves (increased at 24 or 26 µW/g and decreased at 2.4 or 2.6 µW/g) and longer exposures (21 h versus 2 h). Another study by Mashevich et al*.*^29^ reveals that continuous 72 h exposure of human peripheral blood lymphocytes to EMFs of 830 MHz waves, with SAR ranging from 1.6 to 8.8 W/kg lead to SAR dependent aneuploidy with specific abnormalities on chromosome 17. However, in vitro exposure of human blood lymphocytes for only 2 h to short pulses of 2450 MHz, at an average power of 5 mW/cm^2^ ^30^ showed no significant DNA damage as assessed by alkali comet assays. No signs of genotoxicity were found when total human blood leukocytes were in vitro exposed for 24 h either at a continuous or a pulsed-wave 1.9 GHz EMF with a SAR ranging between 0.1 and 10 W/Kg ^31^. Absence of significant DNA damage response in human blood lymphocytes was also reported by a study by Stronati et al.^32^ in which blood specimens were continuously exposed for 24 h at a Global System Mobile Communication generated EMF of 935 MHz with a SAR of 1 or 2 W/Kg ^32^. Similar negative results with respect to EMF induced DNA damage was reported in a study by Hook et al.^33^ with cultured Molt-4 human lymphoblasts exposed for 24 h to four types of frequency mobile network modulations around 815–850 MHz with SAR values ranging from 2.4 to 3.2 W/Kg ^33^.

In our work we test the effects of in vitro irradiating V(D)J recombining pre-B cells with very low doses of RF EM waves. RAG stimulation is obtained either mimicking a pre-BCR stimulus with AKT inhibition, or with a stress inducible Abelson (Abl) kinase inhibitor response via STAT5 phosphorylation inhibition. For both stimuli, near 950–1000 MHz RF EMF cell irradiation, in the absence of detectable DNA DSBs, causes a four-fold reduction in recombination levels in exposed pre-Bs versus that assessed in non-irradiated cells.

## Results

### Design and specific experimental conditions used to assess *Ig k* locus rearrangements

**Figure 1:**
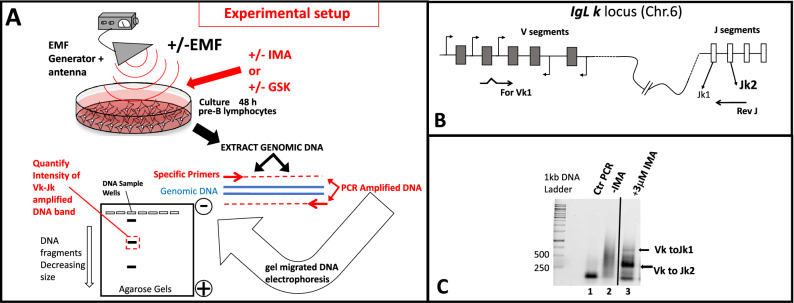
(**A**) Schematic depiction of the flow chart of the experimental design. Murine A-70 vAbl pre-B cells grown with or without exposure to Electromagnetic field influence(EMF), in the absence or presence of RAG stimulation either by Mesylate of Imatinib (IMA) or by GSK-690693(GSK), are harvested and their genomic DNA subjected to a two-steps PCR recombination assay identifying Vk to Jk rearrangements at their *IgL kappa* loci. The electrophoretically separated recombination products (Vk-Jk) are quantified by densitometry to assess the extent of locus rearrangements influenced by EMF. (**B**) Shematic configuration of *IgL k* kappa locus on Mouse chromosome 6, and the positioning of the primers used in the assay. (**C**) PCR reactions electrophoretically separated in agarose gel stained with Sybr green identify the recombined products (arrows show Vk to Jk1 and Vk to Jk2) in lane 3 versus, control reactions lane 1 without genomic DNA, lane 2 templating genomic DNA from uninduced cells (in germline configuration). Such recombination amplified reactions are then used for densitometry quantifications. The entire gel from which (**C**) was cropped displaying amplifications (Vk to Jk response) from cells treated with a wide range of increasing IMA concentrations , is shown in Supplemental Material Fig. 3Sa.

**Figure 2:**
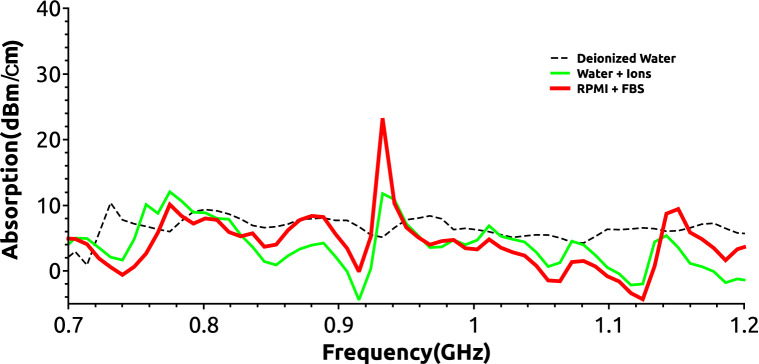
Absorption spectra measurements of filtered deionized water (dashed thin black line), tap water (green thick line Water + Ions) and RPMI cell culture medium with 10% fetal calf serum (FBS)(red thick line RPMI + FBS) All measurements were done using a Keysight-AGILENT-HP N9935A spectrum analyzer as described in “Methods” section.

**Figure 3:**
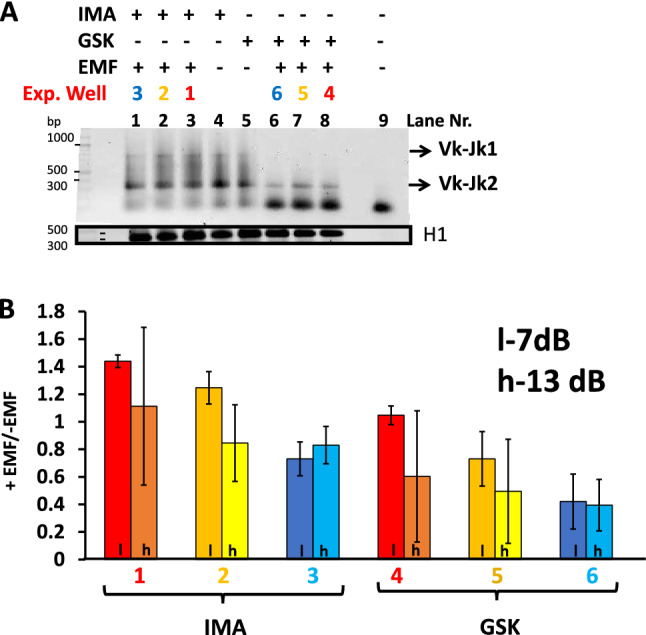
A two steps PCR recombination assay is used to identify Vk to Jk1 or Vk to Jk2 rearrangements from pre-B cells upon RAG induction with Imatinib or GSK. (**A**) A Sybr Green stained 1.5% Agarose TBE gel in which the recombination PCR reactions templated with initial 1:5 dilutions of genomic DNA extracted from each distinct cell treatment lot (2 × 10^6^ cells) are electrophoretically resolved. The cells were either unexposed (gel reaction lanes 4 and 5) or subjected for 48 h to 1 GHz EMF irradiation (lanes 1 to 3 and 6 to 8) with the generator setting at 13 dBm. The color code designating the positions of exposed EMF (exp.Well) wells in the dish is the same with the one used in supplementary Fig. 1Sb. Last lane (9) of the gel, -DNA control reaction. The bottom black box (cropped from a distinct gel) displays Hisone H1 PCR reactions templated with the same amount of genomic DNA as the recombination reactions above(control genomic DNA). (**B**) Identified Vk to Jk2 recombined products were quantified from scanned gels corresponding to PCR reactions from cells +/− Irradiation and the calculated ratios of band intensities expressed + EMF/−EMF(irradiated/nonexposed) for each well (color code consistent with that shown in Fig. 1S). The histograms represent the average values of three independent quantified experiments. EMF-Electromagnetic Field, Recombination pharmacological stimuli (Imatinib, IMA) versus (GSK-690693, GSK). H1, histone H1 control reaction PCR reactions. Darker font histograms correspond to lower 7 dBm (l) and brighter to higher 13 dBm(h) generator power settings.

Our study tests how gene recombination levels are influenced by exposure to EMFs with distinct emitted frequencies and power levels (dose–response). In vitro grown vAbl transformed murine pre-B cells stimulated to recombine V(D)J are exposed to a broadband (0.8–3 GHz) emission antenna which broadcasts an EMF from a RF generator (Fig. 1A upper region). For all experiments we standardized our cellular growing conditions to control irradiation parameters (see Supplemental Material section S1 and Fig. 1Sa and b). RAG expression and V(D)J recombination can be induced in vAbl transformed pre-B cells(differentiating them in small pre-B cells) upon stimulation either with an Abl tyrosine kinase inhibitor imatinib(mesylate of imatinib)(IMA)^34,35^(Supplemental Material Fig. 1Sb growing dish wells 1, 2 and 3), or with an AKT inhibitor GSK-690693(GSK)^19^(wells 4, 5 and 6 , Fig. 1Sb). Whereas IMA induces RAG by inhibiting vABL-1 tyrosine kinase via a stress-inducible GADD45α action^17,34,35^, GSK acts as AKT inhibitor, reducing NF-kB and FOXO1 inhibitory phosphorylation (by CDK4) thus, mimicking a physiologic pre-BCR stimulation^19^ (see Supplemental material section S2). Time course experiments with RAG induction in vAbl pre-B cells using both drugs show maximal RAG1 levels after 36 h of stimulation (see Supplemental material S2 and Fig. 2Sa and b). Using this finding, after 48 h post drug induction (to allow recombination), all synchronized cultured cells were harvested and their genomic DNA extracted. A previously described two-steps nested PCR (polymerase chain reaction) which assesses the recombination extent taking place at *Igk* kappa light chain locus (chromosome 6, locus schematic and primer positions shown in Fig. 1B), is templated with the equivalent genomic DNA extracted from 2 × 10^6^ cells from each tested culture set^36,37^. In the absence of V(D)J recombination (control reactions with no stimulation Fig. 1C lane 2) the variable region V and J segments in germline configuration are too far apart on the chromosome to yield appropriate amplification products. The PCR amplification products obtained only from recombined templates (Fig. 1C lane 3) are separated after electrophoretic migration on 1.5% agarose gels and visualized after fluorescent staining with SYBR green (schematic lower drawing Fig. 1A, and gel scan Fig. 1C). This typical nested PCR reaction reports *k* locus recombination events with two detectable products; the predominant one Vk-Jk2 of 280 bp (95%) and Vk-Jk1 of 700 bp (5%)^36^ (Fig. 1C lane 3). Densitometric quantifications of the DNA Vk-Jk2 recombination products allow us to assess the EMF influence on recombination (Fig. 1A lower drawing). A dose–response (recombination) effect obtained with increasing IMA concentrations in 48 h stimulated pre-B cells is shown in supplementary Fig. 3Sa, gel and quantified data from three such experiments shown in Fig. 3Sb histograms. The lowest drug concentration (3 µM for IMA and of 10 µM for GSK,) for which maximal recombination effects are obtained, is used for each drug in our irradiation assays. For linear range quantifications of the image scans each reaction uses genomic DNA template at least at three distinct dilutions from the cellular extraction stock solution and the final result may be reported as an average of the three quantified products values corrected by the histone H1 band intensity of the corresponding sample. In Supplemental material in Fig. 3Sc an 3Sd a set of nested PCR reactions templated with serial dilutions of input genomic DNA from IMA stimulated cells, followed by quantitation of the signal are shown to illustrate that the assay responds linearly in its amplified Vk-Jk2 band intensity.

### EM wave absorption spectrum of the cell culture medium

We measured how the EM waves with frequencies ranging from 700 to 1224 MHz are absorbed by the fetal bovine calf serum supplemented cell culture medium (RPMI + FBS in Fig. 2) in which the pre-B cells are cultured. For comparison only absorption measurements were also performed for deionized water (conductivity < 5 µS/cm), and for ions containing unfiltered tap water samples (see Methods Water + ions, Fig. 2). The measurements were done using a setup in which an emission and a reception horn antenna were spaced 1 m apart with the liquid sample container positioned in the vicinity(1 cm) of the later (see Supplemental material Fig. 4S). The emission antenna was connected to a generator and signals from the receiver antenna were collected and recorded by a standard spectrum analyzer. In Fig. 2 are presented the background corrected absorption spectra per 1 cm width of each liquid sample measured. A well-defined absorption peak is observed at 938 MHz for the RPMI + FBS medium sample which is twice as large as the others measured at this frequency. All samples have similar absorption values for the rest of the tested spectral frequencies. This finding is important since the range of frequencies (720 MHz, 850 MHz, 950 MHz, 1 GHz and 1.2 GHz) to be used for cell irradiation centers our window of exposure between 950 MHz and 1 GHz, proximal to the maximal culture medium absorption peak.

**Figure 4:**
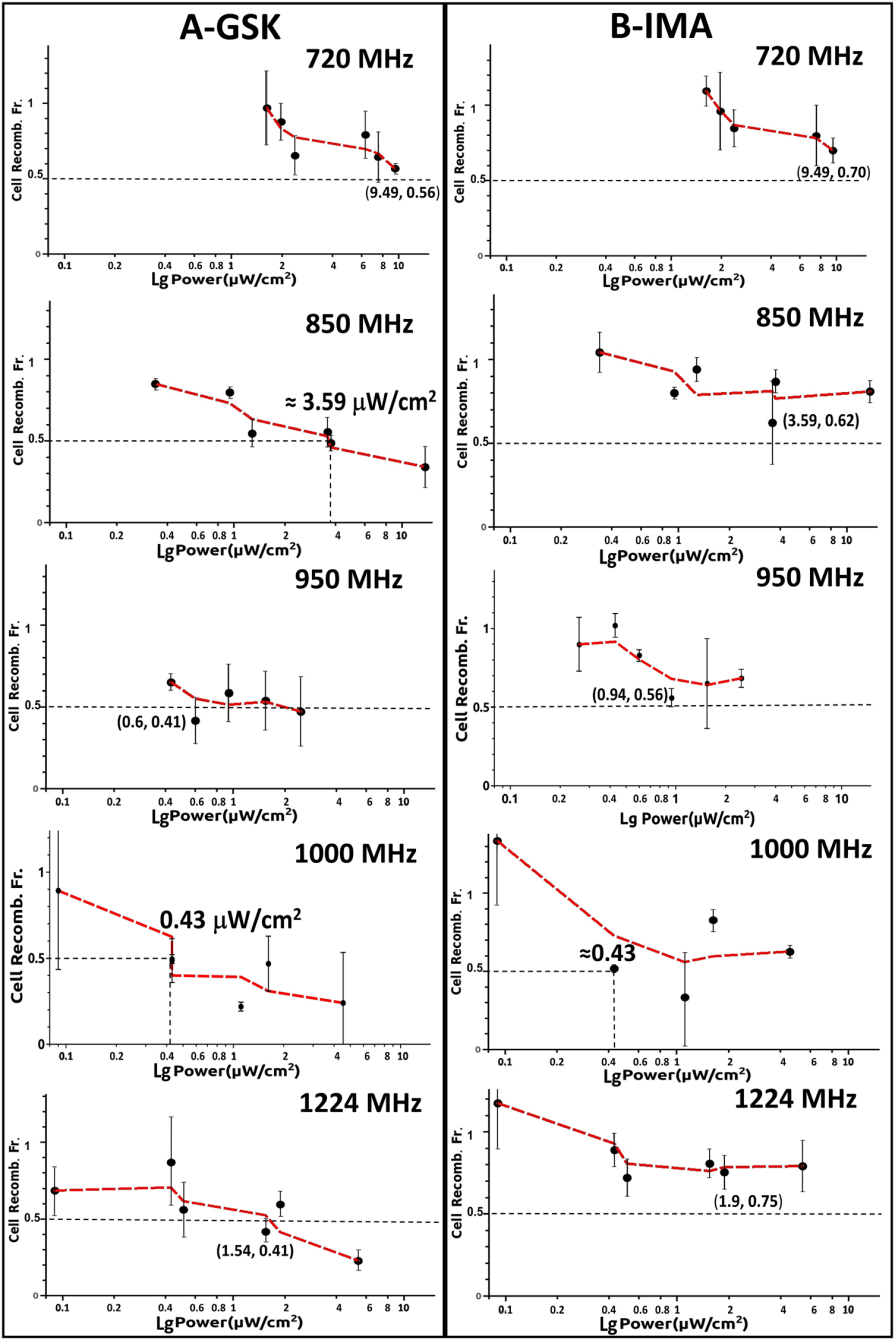
The EMF power dose-cell recombination response curves at 720, 850, 950, 1000 and 1224 MHz for both types of pharmacological agents stimulating RAG expression (**A**) (GSK-690693, GSK), and (**B**) (Imatinib, IMA). Cell Recomb. Fr. expresses the ratio values of measured Vk-Jk2 recombination quantified from cells grown in + EMF/-EMF (irradiated/non-exposed) conditions. Bottom abscissa displays logarithm of S power flux density values (Power µW/cm^2^) measured around the emitting antenna inside the CO_2_ 5 vol%, and 95% water humidity incubator air conditions, expressed as a single range in all panels(logartithmic scale). The black dotted line denote a level of EMF induced two-fold recombination reduction (Cell recomb. Fr. = 0.5), whereas when this level is not reached in the experiment the coordinates of the lowest obtained Cell Recomb. Fr. are given. The red dotted line connecting markers is just a Moving Window Average line which accounts for the average between successive data points displaying the trend of data variation. The error bars represent standard deviation (SD) values from three independent experiments.

**Figure 5:**
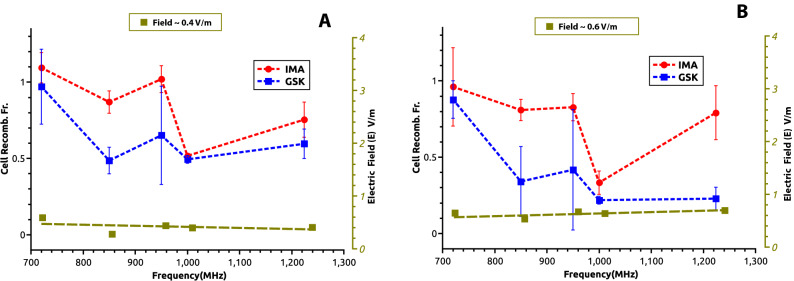
Variation of cell recombination fraction (+ EMF/-EMF) with the field irradiation frequency shown in each panel for a constant receiver EMF electric intensity field E calculated in the cell culture medium. (**A**) EMF electric field intensity E 0.4 V/m, (**B**) EMF electric field intensity E 0.6 V/m. The pharmacological agents stimulating RAG expression GSK-690693, GSK-blue, and Imatinib, IMA-red. The pale green dotted line shows the relative constant distribution of measured electric field as a function of frequency. The error bars represent standard deviation (SD) values from three independent experiments.

To test how the cell growing medium affects the electric intensity of the exposing fields, EMF electric flux density (D displacement) measurements were made inside the incubator for each mentioned frequency, in the absence or presence of culture medium in the culture plate (Supplemental Material S3 and Fig. 5S). Values greater than one of the **D**_**m**_**/D**_**air_inc**_ (1.8–1.95) ratios measured between 750 and 1000 MHz (Supplemental material section S3 and Fig. 5Sc) show in this range, the complete RPMI + FBS cell growing medium selectively potentiates the developed fields.

### EMF irradiation effects on V(D)J recombination in v-Abl pre-B cells

Murine vAbl pre-B cells were grown under normal conditions or stimulated either with 3 µM IMA or with 10 µM GSK in the presence/absence of an antenna which emits a generator controlled EMF from waves of 720 MHz, 850 MHz, 950 MHz, 1 GHz, 1.224 GHz each with 7 or 13 dBm output power setting. For all exposures, the antenna was held at 2.4 cm above the composite 6 wells plate as depicted in Supplemental material Fig. 1Sb (lower profile drawing) consistently keeping it in the same location with respect to the incubator walls (Supplemental material S1 and Fig. 1S). Cells were grown +/− EMF constant continuous exposure for 48 h with +/− IMA or +/− GSK. In Fig. 3A is shown a gel with resolved reactions either from nonexposed cells (lanes 4 and 5) or from cells continuously subjected for 48 h to the influence of 1 GHz fields (gel for generator set at 13dBm-h), with both RAG induction treatments (plate Exp. wells IMA 1, 2, 3 and GSK- 4, 5, 6 with color code shown in Supplemental material Fig. 1Sb). Visually one can see, a reduction of Vk to Jk2 recombination products obtained in reactions from irradiated cells versus those from similarly drug induced, non-irradiated cells (see Fig. 3A compare lane 4 non-irradiated to reactions in lanes 1–3 exposed for IMA, and lane 5 unexposed to lanes 6–8 from irradiated GSK stimulated cells). The irradiating effects are most pronounced in the plate wells closest to the actively emitting antenna elements (λ/2 for 1 GHz waves use as main element the 15 cm one located near wells 3 and 6 (Supplemental material Fig. 1Sb) hence, recombination reduction for plate Exp. wells 3, 6 > 2, 5 > 1, 4 or correspondingly gel lanes 1, 6 > 2, 7 > 3, 8). The value of the calculated ratios between recombination Vk-Jk2 PCR band intensities obtained from irradiated/non-irradiated(+ EMF/-EMF) cells for all tissue culture wells are shown as histograms in Fig. 3B. Values less than one show specific Vk-Jk2 recombination reduction associated with EMF irradiation.

Similar experiments were performed with EMF exposures at 720, 850, 950, 1000 and 1224 MHz (each frequency centers on a different antenna element), generator setting either at 7 dBm or 13 dBm. To display a wider palette of EMF dose exposure values we summed up the data from all of the wells in Fig. 4 which displays cell Vk-Jk2 recombination Fractions(+EMF/−EMF -ordinates), against logarithm of measured irradiating power flux density S values (µW/cm^2^-abscissas) at each location. Each row of the two panels is for a distinct frequency with panels for each drug located on the same column: Fig. 4A(GSK- left) and B(IMA- right). Consistently all diagrams show power dependent reduction in cellular Vk-Jk2 recombination. S values into the emitting antenna were calculated from antenna recorded voltages, circuit impedance, and antenna constructive elements dimensions and reflect S in the air inside incubator, surrounding the involved culture well. In each panel with dotted black lines we pointed the EMF power dose required to induce a two-fold Vk-Jk2 recombination reduction from that of the non-irradiated lot (+EMF/−EMF 50% reduction shown as 0.5 ratio for Vk-Jk2, Cellular Recombination Fraction). In Fig. 4 when 50% recombination reduction (exposed versus non-irradiated cells) is not reached, the minimal recombination ratios obtained and their inducing S levels are shown in parenthesis. The most remarkable finding of our study is that even for such a small window of frequencies (between 720 and 1224 MHz), the power dose–response effect is dramatically influenced by the frequency of the irradiating EMF. If at 720 MHz one reaches a 0.56/0.70 maximal recombination reduction for 9.49 µW/cm^2^ exposure, similar reduction in recombination effects are obtained at 950 MHz and 1 GHz with only 1/15th respectively 1/20th (0.63 or 0.43 µW/cm^2^) the power used at 720 MHz. The power dose-cell recombination response curves at 950 MHz and 1 GHz EMFs show by far the most accentuated measured effects (for both drugs). For GSK at 1 GHz irradiation, an almost four-fold decrease in V(D)J recombination (from 0.90 to 0.22) is observed over a moderate increase in S exposure from 0.1 to 4.53 µW/cm^2^ (see second from the bottom panel in Fig. 4A GSK 1000 MHz). Both curves in Fig. 4 for 1 GHz display an abrupt recombination decrease at a small increase in S (0.25–1 µW/cm^2^) after which the cellular effect plateaus out over a larger window of higher exposure power S values (1–4.5 µW/cm^2^). To emphasize the influence of EMF frequency Table 1 shows how recombination fractions (+EMF/−EMF) vary at a relatively constant **≈** 1.5 µW/cm^2^ irradiating power flux density S exposure level for all tested EMF frequencies. At this small irradiating power no effect is detectable at 720 MHz, whereas at 950 MHz a two-fold recombination reduction is measured reaching almost three-fold recombination inhibiton at 1 GHz.

**Table 1:** Lists the measured cell recombination fraction (+EMF/−EMF) at a relative constant power flux density S value of 1.5 µW/cm^2^ for all tested frequencies.

Fraction recombination +EMF/−EMF (EMF at S ≈ 1.5 µW/cm^2^)		
Frequency (MHz)	Response stimulus	
	GSK	IMA
720	0.97 ± 0.2	1.09 ± 0.1
850	0.56 ± 0.1	0.8 ± 0.1
950	0.53 ± 0.2	0.65 ± 0.3
1000	0.38 ± 0.1	0.46 ± 0.3
1224	0.41 ± 0.1	0.8 ± 0.1

.

To circumvent the cellular growing medium polarization effects (which significantly change polarity at 720 MHz and above 1100 MHz, Supplemental material 3S and Fig. 5Sc) or its enhanced wave absorption at 938 MHz (Fig. 2), we intentionally represented in Fig. 5 the recombination fractions for two constant electric field intensity E exposure values, measured inside the medium; one of 0.4 V/m (Fig. 5A.) and the other of 0.6 V/m (Fig. 5B). The approximative intensity of the emitted electric field was calculated in the cell medium from the measured electric flux density (**D**_**m**_ displacement) values^38^ described in the previous section, and averaged for the central plate well. For both E values and both pharmacological stimuli (IMA-red and GSK-blue) the most accentuated plots concavities (maximal irradiation induced recombination reduction effect), correspond to 950–1000 MHz. At both E values represented in Fig. 5 the recombination ratios are unaffected by EMFs at 720 MHz. In contrast, at 1000 MHz, a two-fold reduction is observed for the 0.4 V/m EMF intensity, and a three (IMA) to four-fold (GSK) decrease is measured at the stronger 0.6 V/m field exposure. The electric fields dose exposures -recombination reduction effects in Fig. 5 and those reported for EMFs power dose exposures in Fig. 4 are qualitatively similar. These data strongly suggest that exposure even to very low irradiation doses from specific 900–1000 MHz radiofrequency waves dramatically affect in irradiated pre-B cells the efficiency of V(D)J recombination at their Ig kappa locus.

### Histone H2AX phosphorylation shows no detectable DNA DSB damage cell response in EMF exposed pre-B cells

**Figure 6:**
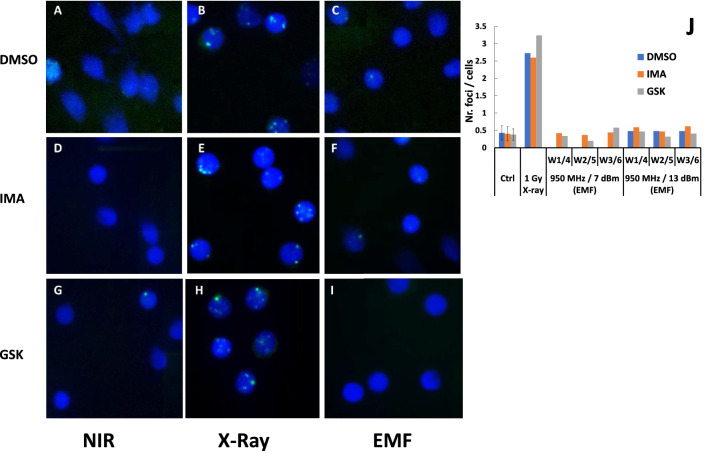
(**A**–**I**) Immuno-fluorescence detection of γH2AX phosphorylated histone foci in pre-B cells exposed to EMF or X ray irradiation. The γ H2AX foci are shown in bright green—γ-H2AX, whereas DNA staining is in blue – nuclei. (**A**) Control solvent (DMSO) treated cells Nonirradiated (NIR); (**B)** Control (DMSO) treated 1 Gy X-ray irradiated cells(X-Ray); (**C**) Control (DMSO)treated EMF exposed (waves at 950 MHz, with emission generator power setting set at 7 dBm-EMF); (**D**) IMA treated NIR; (**E**) IMA, X-ray; (**F**) IMA, EMF; (**G**) GSK, NIR; (**H**) GSK, X-ray; (**I)** GSK, EMF. (**J)** Number of counted foci per /cell represented as histograms. W1/4 refers to growing plate wells 1 and 4, W2/5 wells 2 and 5 and W3/6 wells 3 and 6 equivalent positions with respect to which cells were EMF irradiated, with generator power setting set at 7 dBm and 13 dBm. GSK is cells treatment with 10 µM GSK-690693, IMA their treatment with 3 µM Imatinib. ANDOR camera assisted by IQ Live Cell Imaging software and foci analysis with Imaris for Cell Biologists software (both from OXFORD Instruments).

We asked whether the observed EMF irradiating effects on V(D)J recombination are due to DNA damage and presence of unrepaired DSBs. Impairment of DNA integrity can be assessed by the extent with which irradiation induces H2AX histone phosphorylation (γH2AX), a process associated with DNA DSBs and their intranuclear repair. The nuclear γH2AX repair foci are the fairest indication that the NHEJ DNA repair machinery acts properly in these cells repairing DSBs caused by any DNA lesion-causing agent^39,40^. We grew cells under similar stimulation (+/− IMA, +/− GSK) and +/− EMF irradiation conditions (7 dBm or 13 dBm generator power settings at 950 MHz) with those described above but instead of extracting DNA, the harvested cells were fixed and doubly stained: (a) with Hoechst 33342 dye (for nuclear total DNA staining in blue) and (b) immunofluorescently with anti γH2AX antibodies yielding a Cy2 green fluorescence which identifies DNA DSBs repairing γH2AX foci^40^(see Methods). As a DNA DSBs control an extra lot of cells were either noninduced o similarly drug treated but instead of EMF they were subjected to a quick 1 Gy, X ray irradiation dose prior to their harvest. Nine immunofluorescent images are shown in Fig. 6 A-I where blue contours show the cell nuclei and the green dots the DNA DSBs repairing γH2AX foci from: cells treated with +/− DMSO solvent control, +/− GSK, +/− IMA, +/− EMF set at 950 MHz, 7dBm exposure and the control lot of cells exposed to 1 Gy X ray. Such foci were also counted and their number reported per cell to a number of total 100 counted cells gathered from more than twenty successive field views for each experimental lot (shown as histograms in Fig. 6J for both 7 dBm and 13 dBm generator power settings). 1 Gy dose X ray irradiated cells are shown in Fig. 6B control with DMSO solvent, E with IMA, H with GSK and in 6 J the corresponding foci/cell counted histograms. All images (Fig. 6B,E,H) and the quantified histograms from X ray irradiated cells show similar and considerable DNA DSB lesions with consequent accumulation of γH2AX repair foci, regardless of the chemical stimulus used. On the contrary, the long 48 h EMF exposure experiments do not show signs of detectable unrepaired DNA DSB damage (Fig. 6C DMSO solvent, F with IMA and I with GSK, and counted foci/cells in Fig. 6J), above the background level of non-irradiated control cultures (Fig. 6A,D,G and ctrol. histograms in Fig. 6J). Exposing for 48 h cells to EMF , regardless of drug treatment, does not seem to inflict significant/ cumulative unrepaired DNA DSB lesions, (unlike those caused even by mild quick irradiation with 1 Gy dose of X rays). Only such DNA injuries could have caused a detectable accumulation of repairing γH2AX foci at the time of their harvest. Indirectly, these results suggest that the significant EMF induced reduction in pre-B cells recombination reported in Figs. 3B, 4, 5 and Table 1 is probably not caused by an enhanced level of accumulated unrepaired DNA DSBs.

## Discussion

V(D)J recombination the central process in lymphocyte development physiologically generates DNA DSBs during its course, when cells become susceptible to external sources of DNA damage^5^. Our work tests how pre-B lymphocytes exposure to low dose EMFs of frequencies ranging from 720 MHz to 1.2 GHz, used in utilitarian purpose telecommunication, affects the efficiency of their *Igk* loci rearrangements. First, we established a setup to control the EMF developed inside the cellular growing medium in a typical cell culture incubator. Cultured pre-B cells synchronously recombining V(D)J were EMF exposed during a 48 h window, which starts with RAG expression and ends with the NHEJ DSBs DNA repair^5^. A nested PCR assay is then used to study the cellular EMF irradiation gene effects.

The measured data in Figs. 3B, 4, 5 and Table 1 consistently show, EMFs cause a dose dependent reduction in V(D)J recombination in the irradiated pre-B cells, with similar effects for both RAG inducing stimuli (IMA or GSK) used. The magnitude of effects is tightly determined by the EMF frequency. A two-fold reduction in Vk-Jk2 recombination at *Igk* locus narrowly can be obtained by an emitted S power value of 9.49 µW/cm^2^ at 720 MHz (Cell Recomb. Fr. 0.56 for GSK and 0.7 for IMA), whereas this effect may be achieved by a field developing one twentieth of that S dose at 1000 MHz (0.5 Cell Recomb. Fr. for both drugs at 0.43 µW/cm^2^) (Fig. 4). The recombination reduction although observed for all EMFs tested, seems to be maximal for 950 and 1000 MHz waves, a small domain where the serum containing cell culture medium displays maximal EMF absorbance (Fig. 2), and augments by its molecular polarization the EMF electric intensity (supplementary Fig. 5Sc). We measured EMF local antenna emissive S values only in the incubator air surrounding the cell culture plate. Despite this limitation we measured and calculated the average irradiated electric field intensity E, inside the culture medium. The maximal effects were measured at 950 and 1000 MHz, where *Igk* recombination levels for an EMF of E 0.4 V/m are only half (Fig. 5A), or for one of 0.6 V/m E a quarter of those reported for same E values at 720 MHz (Fig. 5B). E dose effects parallel the frequency dependency described for the antenna emitted power dose S. However, the cell medium electric properties mentioned above, (increased absorbance and polarization between 900 and 1000 MHz), cannot account for the frequency results shown in Fig. 5 for irradiations at constant electric field E values. Besides such intrinsic medium properties there must be also a major EMF frequency direct influence on the cellular components linked to recombination.

Various wireless network service providers use for mobile phone communication frequencies ranging between 700 and 2100 MHz. At 1 cm distance, during outgoing calls the measured emitted field E intensities vary with +/− 5–15% from the 41.25 V/m ( recommended ICNIRP value) with cell phone models, whereas their maximum output recorded power levels for a GSM1800 net varies between 0.25 and 1 W^41,42^.

We assessed if the low dose 48 h EMF irradiations cause DNA DSBs and detectable γH2AX repair foci in exposed cells. From the levels of detected γH2AX repair foci of the EMF irradiated pre-B cells we could not reveal in exposed cells above background DNA DSBs repair activity (Fig. 6 compare panel A with C, G with I, and histograms in Fig. 6J). Using chromatin immunoprecipitation (ChIP) Savic et al.^43^ show considerable γH2AX accumulation near Jk5 in IgK locus after 24 h post STI571 (Imatinib) treatment of pre-Bs, but a dramatic more than two-fold decrease in γH2AX detection as cells were kept from 24 to 48 h post STI571 treatment^43^. We could not detect above background γH2AX foci levels in IMA or GSK treated cells after 48 h culture growth. This could be due either to a considerable post RAG DSBs repair recovery, or to a reduced sensitivity of our immunofluorescence assay (less sensitive than ChIP in detecting γH2AX). The onset of DNA DSBs either prior to or during pre-B cells maturation inhibits *rag1*, *2* transcription^44^ and reduces the levels of *Igk* locus rearrangement events^45^. These cellular stress effects are caused by ataxia teleangiectasia mutated (ATM) kinase either via NF-kB, FOXO1 signaling^44,46^ or via GADD45α inhibition^45^, both pathways directly targeting *rag* genes transcription levels. If very few EMF induced DSBs (below those detectable by γH2AX foci assay), or breaks already repaired before our cell harvests could have reduced RAG expression in our experiments(via ATM kinase) this could explain our observed reduced recombination effects. We used in our experiments two RAG induction stimuli, IMA sensitive to ATM kinase via GADD45α inhibition ^44–46^ and the second GSK690693 AKT-inhibitor insensitive to this signaling pathway^19^. If very few EMF induced DNA DSBs would have reduced RAG expression prior to, or during drug action, one would have expected experiments to show a more accentuated recombination reduction for IMA than that obtained for GSK treatment. Instead, the experimental data in Figs. 3B, 4, 5 and Table 1 show for both drugs very similar EMF induced reduction of *Igk* loci rearrangement levels(if not even slightly more pronounced reduction for GSK). IMA although a more potent RAG inducer than GSK has the disadvantage that post recombination blocks cells in Go phase preventing further their division^36,47–49^. On the contrary, the AKT inhibitor GSK-690693 not only is a weaker RAG induction stimulus (closer to the one physiologically occurring in small pre-B cells)^50,51^ but also enables cells to divide prior to and after *Igk* loci rearrangements and protect their progress to the next stage of development^19^. Because our PCR assay intentionally uses the amount of templating genomic DNA from the same number of 2 millions harvested cells, replication would have “diminished the EMF recombination reduction” in GSK treated cells in contrast to those incubated with IMA (the later, on the contrary, “freezes” the EMF effect on BCL2 maintained survivors). As pointed earlier, in treated cells, both drugs show very similar EMF induced reduction of rearrangements in treated cells. Although we cannot fully refute that the observed EMF recombination effects may have been caused in irradiated pre-B cells by undetectable DNA DSBs via ATM, the line of evidence gathered from our experiments in Figs. 4, 5, 6 and the arguments presented above for the comparative IMA/GSK treatments make this mechanism a less likely candidate for their account.

Indirectly our work addresses the longstanding question of how innocuous low dose EMF irradiation from our telecommunication devices may be and whether it may affect the immunity of our organisms. It remains only to our speculation to extend the observed recombination effects induced by small EMFs from an in vitro culture system to the in vivo situation on the ability of irradiated B cells to elicit an unaltered antibody response to antigen challenge.

## Methods

### Materials

DNA oligonucleotides were purchased from Life Technologies and IDT DNA: Vk degenerate primer 5′ GCTGCAGSTTCAGTGGCAGTGGCAGTGGRTCWGGRAC 3′ where S is G or C, R is A or G, W is T or A, Jk2-1 primer 5′ CAAAACCCTCCCTAGGTAGACAATTATCCCTC 3′ and Jk2-2 primer 5′ GGACAGTTTTCCCTCCTTAACACCTGATCTG 3′. For Histone H1 gene control amplifications the following primers were used: H1fw 5′ GGCTGCTATCCAGGCAGAGAAGAACCG 3′, H1rv: 5′ GCTTTGGAGGCGCCTTCTTGGGCTTG 3′.

*Murine pre B cells* transformed with Abelson virus (v-Abl preB, A70 line, that harbor a Eµ-Bcl2 transgene) were a kind gift from Barry Sleckman Duke University^47^. The cells were maintained in RPMI 1640 medium, supplemented with 10% FBS (both from GIBCO), 50 µM 2-mercaptoetanol and induced at 0.5 × 10^6^ cells/ml density either with 3 μM Imatinib Mesylate (IMA) (SIGMA-ALDRICH) or with 10 µM GSK-690693(GSK)( GLAXOSMITHKLINE, SELLECK-chem) in solutions with 0.1% DMSO. After 48 h the cells were collected and analyzed using the nested PCR described below.

*Pre-B Cells irradiation* was performed with a 1 Hz–1.224 GHz, 13 dBm radiofrequency generator (Hameg Instruments 1 Hz–1.2 GHz programmable synthesizer HM8134-3, used throughout our study as emission generator) using a broadband irradiating 800 MHz–3 GHz LTE ATK-LOG ALP logarithmic antenna, in a regular CO_2_ incubator (SANYO Electric Co. MCO-17AIC), with CO_2_ 5 vol. %, and 95% purified water humidity. Cells were grown at 37 °C in 5 ml medium in standard six flat bottom wells (16.8 ml capacity) polystyrene lidded plates (Corning Costar), which were always positioned in the same place with respect to the incubator walls (in the center of the incubator, see Supplemental material Fig. 1Sa) and the emission antenna (antenna central guiding label positioned midway between wells 3 and 6 at 2.4 cm above the mid plane of the plate, see supplementary Fig. 1S). Two parallel sets of experiments were performed with wells 1, 2, 3 containing cells stimulated with 3 µM IMA, whereas wells 4, 5 and 6 cells were stimulated with 10 µM GSK (Fig. 1SB).

### Two steps nested PCR reactions for K locus recombination

Template DNA was prepared for PCR using a modified technique developed by Schlissel^37^. Pre-B A-70 v-Abl cells were harvested after 48 h incubation with IMA^36,47^, GSK^19^ or unstimulated. Cultured cells (2 × 10^6^–2 millions) were pelleted for 15 s in a microfuge, washed once in PBS(phosphate saline buffer pH 7.2), resuspended in 200 µl PCR lysis buffer (10 mM Tris pH 8.4, 2.5 mM MgCl_2_, 50 mM KCl, 200 µg/ml gelatin, 0.45% NP40, 0.45% Tween-20 (CALBIOCHEM), and 60 µg/ml Proteinase K(Boehringer), and incubated at 56 °C for 3 h followed by 15 min at 95 °C. Dilution of templates was done with PCR lysis buffer without Proteinase K. Two successive PCR amplifications were done in a final volume of 50 µl containing 2 to 5 µl template DNA, 10 mM Tris–HCl (pH 8.4; at room temperature), 50 mM KCl, 2.5 mM MgCl_2_, 200 µg/ml gelatin, 0.2 mM of all four dNTPs (all from ThermoFisher scientific), each oligonucleotide primer at 0. 4 µM (20 pmol each primer per reaction), and 1 U TAQ DNA polymerase (GoTaq PROMEGA) in nested reactions. First step PCR reactions for 25 cycles use Vk, and Jk2-1primers. In the second step various dilutions (from 4 µls 1:100 dilution of first PCR to 0.5 µls of the first undiluted PCR) are individually used to template the second PCR reactions to which Vk and Jk2-2 primers are added and an additional round of 30 cycles amplification is performed. Cycling steps were: an initial 1 min denaturation at 94 °C, then repeated cycles each, 30 s at 94 °C, 0.5 min annealing at 50 °C, and 1.5 min polymerization at 72 °C. A final additional 5 min extension step was performed at 72 °C ^36,37^. PCR products were resolved on 1.5% agarose gel, stained either with ethidium bromide or Sybr Green (THERMOFISHER scientific) and visualized using the PharosFX system (BIORAD). The bands intensities were quantified using QuantityOne software.

### Kappa locus amplification products analysis

Each Vk-Jk2 product band density of the gel scan image is quantified and the ratio between the densitometry value of the PCR product band detected from cells grown in the presence of EMF and the corresponding one without field exposure (EMF+/EMF−, Cell Recomb. Fr., Figs. 3, 4, 5) reports the changes in V(D)J recombination occurred upon each cell treatment (IMA/GSK). To normalize for DNA extraction levels we performed similar PCR amplifications from the same amount of template DNA using a pair of primers H1fw and H1rv to specifically detect the histone H1 gene.

### γH2AX foci analysis for irradiation induced DNA damage cellular response

Cells were grown under similar conditions with those described above for recombination assays. Additionally, a DNA DSBs control cell lot either uninduced or one for each RAG stimulus (IMA or GSK) was exposed to a quick 20 min X ray cumulative dose exposure of 1 Gray (X-ray irradiation with a slow rate 50-milligray /min with a Mevatron Primus 2D, 6MV, SIEMENS instrument) prior to their harvest. The samples were irradiated at 100 cm distance from the source axis, the field size being of 30 × 30 cm. The dosimetry was performed using a water phantom (1 cm water depth). Symmetry and homogeneity were checked, the dose proved to be homogenous throughout the sample in the used plates. For all treatments, twenty minutes after harvest, instead of extracting DNA, the cells from each individual culture type were separately spread onto clean designated slide sets using a Cytospin Centrifuge. The cells were then fixed with paraformaldehyde, permeabilized with Triton X and then doubly stained with: (a) Hoechst 33342 dye (THERMO SCIENTIFIC) (for their nuclei-DNA total staining in blue) and (b) immunofluorescently with primary unlabeled anti γH2AX antibodies of mouse antigen specificity complemented with secondary Cy2 labeled anti primary source antibodies (rat anti mouse IgG Cy2 detection antibodies-green)(both from SIGMA ALDRICH); to identify in green the DSB repairing γH2AX foci^40^. The slides were examined with a fluorescence microscope (OLYMPUS BX60) with adequate filter for the fluorophores, and images of the nuclei and γH2AX foci recorded with a camera connected to the microscope. The images were analyzed using specific analysis software to quantify the number of foci per each cell treatment type, and morphologically to indicate their level of dispersion or nuclear positioning (see Fig. 6).

*Western blot analysis* for endogenous RAG time course induction in pre-B cells (Supplemental material Fig. 2S) following IMA/GSK treatment was performed as previously described in our work using anti RAG1 and anti RAG2 mouse monoclonal antibodies (gift from Dr. David G. Schatz, Yale University), and control sample purified murine core RAG1(384–1040) and coreRAG2 (1–387) fused to Maltose binding protein (MBP-40kD) which were transiently expressed in co-transfected HEK293T cells^52^ (source ATCC CRL-3216).

*Absorption spectra measurements* were made using two identical broadband (0.8–16 GHz) horn antennas facing each-other and placed at 1 m distance. The measurement subjected sample was placed in close proximity (1 cm) of the receiver whereas the emission antenna (supplementary Fig. 4S a and b), was coupled to the generator. The receiver antenna was connected to a commercial Spectrum Analyzer (Keysight-AGILENT-HP N9935A, 0.1- 9 GHz) on which the received signals were recorded and analyzed. The shown absorption spectra in Fig. 2 were obtained after subtraction of the background spectra with no liquid sample placed in the container in front of the receiver antenna. The deionized water used for measurement has the conductivity < 5 µS/cm, whereas the used unfiltered tap water with ions has the following characteristic measured chemical parameters per liter (l) pH 6.5–9.5, Conductivity < 800 µS/cm, ammonia < 0.5 mg/l, free residual Chlorine < 0.5 mg/l, Fe < 200 µg/l, Mn < 50 µg/l, Al < 200 µg/l, nitrites < 0.5 mg/l, nitrates < 50 mg/l, Borate salts 1 mg/l, Chlorides 250 mg/l, Sulphates 250 mg/l, 65 mg/l calcium carbonate, Hardness < 5degrees (dGH).

## Supplementary Information

Supplementary Information.

## References

[1] Tonegawa S (1983). Somatic generation of antibody diversity. Nature.

[2] Kim MS, Lapkouski M, Yang W, Gellert M (2015). Crystal structure of the V(D)J recombinase RAG1-RAG2. Nature.

[3] Sadofsky MJ (2001). The RAG proteins in V(D)J recombination: more than just a nuclease. Nucleic Acids Res..

[4] Schatz DG, Ji Y (2011). Recombination centres and the orchestration of V(D)J recombination. Nat. Rev. Immunol.

[5] Schatz DG, Swanson PC (2011). V(D)J recombination: mechanisms of initiation. Annu. Rev. Genet..

[6] Herzog S, Reth M, Jumaa H (2009). Regulation of B-cell proliferation and differentiation by pre-B-cell receptor signalling. Nat. Rev. Immunol..

[7] Peschon JJ (1994). Early lymphocyte expansion is severely impaired in interleukin 7 receptor-deficient mice. J. Exp. Med..

[8] O'Shea JJ, Plenge R (2012). JAK and STAT signaling molecules in immunoregulation and immune-mediated disease. Immunity.

[9] Goetz CA, Harmon IR, O'Neil JJ, Burchill MA, Farrar MA (2004). STAT5 activation underlies IL7 receptor-dependent B cell development. J. Immunol..

[10] Yao Z (2006). Stat5a/b are essential for normal lymphoid development and differentiation. Proc. Natl. Acad. Sci. U. S. A..

[11] Cooper AB (2006). A unique function for cyclin D3 in early B cell development. Nat. Immunol..

[12] Jiang Q (2004). Distinct regions of the interleukin-7 receptor regulate different Bcl2 family members. Mol. Cell Biol..

[13] Ramadani F (2010). The PI3K isoforms p110alpha and p110delta are essential for pre-B cell receptor signaling and B cell development. Sci Signal.

[14] Amin RH, Schlissel MS (2008). Foxo1 directly regulates the transcription of recombination-activating genes during B cell development. Nat. Immunol..

[15] Dengler HS (2008). Distinct functions for the transcription factor Foxo1 at various stages of B cell differentiation. Nat. Immunol..

[16] Eijkelenboom A, Burgering BM (2013). FOXOs: signalling integrators for homeostasis maintenance. Nat. Rev. Mol. Cell Biol..

[17] Kuo TC, Schlissel MS (2009). Mechanisms controlling expression of the RAG locus during lymphocyte development. Curr. Opin. Immunol..

[18] Ozes ON (1999). NF-kappaB activation by tumour necrosis factor requires the Akt serine-threonine kinase. Nature.

[19] Ochodnicka-Mackovicova K (2015). NF-kappaB and AKT signaling prevent DNA damage in transformed pre-B cells by suppressing RAG1/2 expression and activity. Blood.

[20] Ochiai K (2012). A self-reinforcing regulatory network triggered by limiting IL-7 activates pre-BCR signaling and differentiation. Nat. Immunol..

[21] Inlay MA, Tian H, Lin T, Xu Y (2004). Important roles for E protein binding sites within the immunoglobulin kappa chain intronic enhancer in activating Vkappa Jkappa rearrangement. J. Exp. Med..

[22] Herzog S (2008). SLP-65 regulates immunoglobulin light chain gene recombination through the PI(3)K-PKB-Foxo pathway. Nat. Immunol..

[23] Lieber MR, Yu K, Raghavan SC (2006). Roles of nonhomologous DNA end joining, V(D)J recombination, and class switch recombination in chromosomal translocations. DNA Repair (Amst).

[24] Marculescu R, Le T, Simon P, Jaeger U, Nadel B (2002). V(D)J-mediated translocations in lymphoid neoplasms: a functional assessment of genomic instability by cryptic sites. J. Exp. Med..

[25] Delimaris J, Tsilimigaki S, Messini-Nicolaki N, Ziros E, Piperakis SM (2006). Effects of pulsed electric fields on DNA of human lymphocytes. Cell Biol. Toxicol..

[26] Phillips JL, Singh NP, Lai H (2009). Electromagnetic fields and DNA damage. Pathophysiology.

[27] Olive PL, Banath JP (2006). The comet assay: a method to measure DNA damage in individual cells. Nat. Protoc..

[28] Phillips JL (1998). DNA damage in Molt-4 T- lymphoblastoid cells exposed to cellular telephone radiofrequency fields in vitro. Bioelectrochem. Bioenerg..

[29] Mashevich M (2003). Exposure of human peripheral blood lymphocytes to electromagnetic fields associated with cellular phones leads to chromosomal instability. Bioelectromagnetics.

[30] Leal BZ, Szilagyi M, Prihoda TJ, Meltz ML (2000). Primary DNA damage in human blood lymphocytes exposed in vitro to 2450 MHz radiofrequency radiation. Radiat. Res..

[31] McNamee JP (2003). No evidence for genotoxic effects from 24 h exposure of human leukocytes to 1.9 GHz radiofrequency fields. Radiat. Res..

[32] Stronati L (2006). 935 MHz cellular phone radiation. An in vitro study of genotoxicity in human lymphocytes. Int. J. Radiat. Biol..

[33] Hook GJ (2004). Measurement of DNA damage and apoptosis in Molt-4 cells after in vitro exposure to radiofrequency radiation. Radiat. Res..

[34] Muljo SA, Schlissel MS (2003). A small molecule Abl kinase inhibitor induces differentiation of Abelson virus-transformed pre-B cell lines. Nat. Immunol..

[35] Wilson MK, McWhirter SM, Amin RH, Huang D, Schlissel MS (2010). Abelson virus transformation prevents TRAIL expression by inhibiting FoxO3a and NF-kappaB. Mol. Cells.

[36] Carmona LM, Fugmann SD, Schatz DG (2016). Collaboration of RAG2 with RAG1-like proteins during the evolution of V(D)J recombination. Genes Dev..

[37] Schlissel MS, Baltimore D (1989). Activation of immunoglobulin kappa gene rearrangement correlates with induction of germline kappa gene transcription. Cell.

[38] Gregson SM, McCormick J, Parini C (2007). Principles of Planar Near-Field Antenna Measurements.

[39] Helmink BA (2011). H2AX prevents CtIP-mediated DNA end resection and aberrant repair in G1-phase lymphocytes. Nature.

[40] Huang X, Darzynkiewicz Z (2006). Cytometric assessment of histone H2AX phosphorylation: a reporter of DNA damage. Methods Mol Biol.

[41] I. C. o. N.-I. R. P. (2020). Principles for non-ionizing radiation protection. Health Phys..

[42] Isabona, J. & Srivastava, V. M. (2017) Cellular mobile phone—a technical assessment on electromagnetic radiation intensity on human safety. *IEEE*, 271–274, Doi: 10.1109/NIGERCON.2017.8281899

[43] Savic V (2009). Formation of dynamic gamma-H2AX domains along broken DNA strands is distinctly regulated by ATM and MDC1 and dependent upon H2AX densities in chromatin. Mol. Cell.

[44] Fisher MR, Rivera-Reyes A, Bloch NB, Schatz DG, Bassing CH (2017). Immature lymphocytes inhibit Rag1 and Rag2 transcription and V(D)J recombination in response to DNA double-strand breaks. J. Immunol..

[45] Steinel NC (2013). The ataxia telangiectasia mutated kinase controls Igkappa allelic exclusion by inhibiting secondary Vkappa-to-Jkappa rearrangements. J. Exp. Med..

[46] Ochodnicka-Mackovicova K (2016). The DNA damage response regulates RAG1/2 expression in pre-B cells through ATM-FOXO1 signaling. J. Immunol..

[47] Bredemeyer AL (2006). ATM stabilizes DNA double-strand-break complexes during V(D)J recombination. Nature.

[48] Hantschel O, Rix U, Superti-Furga G (2008). Target spectrum of the BCR-ABL inhibitors imatinib, nilotinib and dasatinib. Leuk. Lymphoma.

[49] Marinelli Busilacchi E (2018). Immunomodulatory effects of tyrosine kinase inhibitor in vitro and in vivo study. Biol. Blood Marrow Transplant..

[50] Borghesi L (2004). B lineage-specific regulation of V(D)J recombinase activity is established in common lymphoid progenitors. J. Exp. Med..

[51] Grawunder U (1995). Down-regulation of RAG1 and RAG2 gene expression in preB cells after functional immunoglobulin heavy chain rearrangement. Immunity.

[52] Ciubotaru M (2015). The architecture of the 12RSS in V(D)J recombination signal and synaptic complexes. Nucleic Acids Res..

