# Supplementary material for: Radiofrequency EMF irradiation effects on pre-B lymphocytes undergoing somatic recombination
Source: Sci Rep. 2021 Jun 16;11:12651. doi: 10.1038/s41598-021-91790-3 (PMC8208969; doi:10.1038/s41598-021-91790-3)
Supplement: Supplementary file 1 — Supplementary Information. [file 41598_2021_91790_MOESM1_ESM.docx]

**SUPPLEMENTAL MATERIAL**

**Radiofrequency EMF irradiation effects on pre-B lymphocytes undergoing somatic recombination**

**Elena Ioniţă^1,2^, Aurelian Marcu^3^, Mihaela Temelie^1^, Diana Savu^1^, Mihai Şerbănescu^3,4^ and Mihai Ciubotaru^1,2,*^**

^1^Horia Hulubei National Institute for R&D in Physics and Nuclear Engineering, Department of Physics of Life and Environmental Sciences, Măgurele, Ilfov, 077125, Romania;

^2^Colentina Clinical Hospital, Department of Immunology, Internal Medicine, 72202, Bucharest, Romania;

^3^ National Institute for Laser Plasma and Radiation Physics, Center for Advanced Laser Technologies, Măgurele, Ilfov, 077125, Romania;

*** corresponding author: Mihai Ciubotaru, e-mail: mihai.ciubotaru@nipne.ro,

**1S. Standardized in vitro pre-B cells growing conditions for RF EMF irradiation**

A typical six-wells polystyrene cell culture plate was used with respect to which the emission antenna was invariably placed for all experiments in the exact same position in the incubator, above the plate (see irradiation setup scheme in Supplemental Material Fig. 1Sa) with its guiding mark aligned between wells 3 and 6 of the plate underneath (Supplemental Material Fig. 1Sb). In Fig. 1Sa are shown all the coordinates from the antenna guiding mark to the incubator walls, indicating its strict positioning. With respect to the cell culture plate, wells 1 and 4 (red), 2 and 5 (yellow), 3 and 6 (blue) were designed as pair-wise equivalents in terms of field exposure levels (as shown in Fig. 1Sb Supplemental Material), intended for testing two distinct V(D)J recombination inducing stimuli(IMA and GSK) used in the study (see main text). The positioning of the antenna and cell culture plate in the incubator (Supplemental Material Fig.1Sa) was guided by prior pilot electric flux density measurements (see below) for which similar values were obtained among the three reference well positions along the plate (thus minimizing the reflection and standing waves). To obtain inside the probes a wider range of power exposure values we used either 7 dBm or 13 dBm generator power settings for each emitted frequency tested. Cells grown in the absence or presence of EMF were harvested and their genomic DNA was used further to assess recombination levels.

**2S. Time course experiments with RAG induction in v-Abl pre-B cells**

We use in culture murine A70 Abelson virus (v-Abl) transformed pre-B cell lines expressing a modified viral oncogene v- Abl tyrosine kinase (resulted from a gene fusion of the N terminus encoding cellular c-Abl kinase with a fragment of the retroviral gag gene)^1^. The v-Abl kinase constitutively phosphorylates STAT-5 which in turn inhibits RAG expression, mimicking an IL-7R stimulus^2^. These cells represent a valuable model mimicking large pre-B cell type, but growing in absence of IL-7 stimulation. RAG expression and V(D)J recombination can be induced in vAbl transformed pre-B cells(differentiating them in small pre-B cells) upon stimulation either with an Abl tyrosine kinase inhibitor imatinib(mesylate of imatinib)(IMA) ^1,2^(Supplemental Material Fig. 1Sb growing dish wells 1, 2 and 3), or with an AKT inhibitor GSK-690693(GSK)^3^(wells 4, 5 and 6 , Fig. 1Sb Supplemental Material), which mimics a pre-BCR stimulus. IMA induces RAG by inhibiting vABL-1 tyrosine kinase acting through RAS-ERK and STAT 5 phosphorylation^1,2^, and by stimulating stress-inducible GADD45α and FOXO3 both of which bind *rag* promoter and *e rag* enhancer increasing transcription^4^. Besides inducing high non-physiologic levels of RAG, due to STAT5/ cyclin D3 inhibition effects, IMA induces G1 checkpoint cell cycle arrest halting cell division^2,5^. The A-70 vAbl pre-B cells used in our study bear an Eμ enhancer controlled *Bcl2* transgene constitutively expressing antiapoptotic pro-survival levels of BCL2^3,6^. The other pharmacological stimulus used GSK acts as AKT kinase inhibitor, reduces NF-kB and FOXO1 inhibitory phosphorylation (by CDK4) which independent from vAbl pathway, allow *e rag* enhancer activation of rag1,2 transcription. Unlike IMA, GSK stimulation of RAG expression does not interfere with cell cycling, allowing S phase before and after RAG activity^3^.

Time course experiments with immunoblot analysis of v-Abl pre-B cell extracts showing RAG1 expression upon IMA or GSK stimulation are depicted in supplementary Fig. 2S. In both cases RAG1 expression reaches maximal levels after 36 h post-stimulation (36 h is lane 6 in Fig. 2Sa Supplemental Material, IMA stimulation or lane 4 in Fig. 2Sb for GSK induction). One notes in IMA treated cells RAG1 expression reaches considerable levels even after 12 h post-stimulation much earlier than those treated with GSK, a reason for which we consider the later drug to better mimic the small pre-B cells physiologic RAG expression profile. The second wave of RAG activity occurs *in vivo* in the bone marrow B lineage during/after the transition from large pre-B (ckit^-^, CD19^+^, IL-7R^+^, CD43^+^, BP-1^-^ ; light chain germ line configuration) to small pre-B cells(ckit^-^, CD19^+^, IL-7R^-^, CD43^-^, BP-1^+^ ; rearranging light chain locus) within a 40-72 h window prior to their IgM surface receptors display (rearranged light chain)^7-9^.

**3S. Electric flux density (D) measurements characterizing emissive EMF fields inside the irradiation setup.**

To test how the cell growing medium affects the electric intensity of the exposing fields, EMF electric flux density (D displacement) measurements were made for each of the mentioned frequency inside the growing incubator, in the absence or presence of culture medium in the culture plate (Supplemental Material Fig. 1Sa). For high frequency waves (with wavelengths shorter or comparable with one of the dimensions of the incubator) the developed EMFs in an incubator are known to have significant spatial variations over short distances^10^. We have used probe calibrated antennas to measure local EMF variations (with or without cell culture medium) inside the incubator to position in the later experiments the Yagi emission irradiating antenna and the culture plate in a location with minimal EMF variations (Supplemental Material Fig. 5Sa and b). The plate position in the incubator found with the least EMF electric intensity values deviations among the readings of the three wells was chosen as reference for irradiating antenna/plate positioning (shown in Supplemental Material Fig. 1Sa). Secondly, we have measured for each frequency the level of electric flux density D of emitted EMF by a probe irradiating and receiver antenna in the ‘near-field’ zone of each reference positioned well and at distances corresponding to well positions (wells with or without cell culture medium). In Supplemental Material Fig. 5Sc we plotted the average of all three plate wells calculated ratios of measured field electric flux density **D_m_** values in growing medium RPMI+FBS versus those correspondingly measured in its absence in the incubator environment **D_air_inc_**. This ratio is displayed with respect to EMF frequency for each emission generator power setting (7dBm red and 13 dBm, blue). To our surprise the RPMI+FBS complete cell growing medium selectively potentiates the fields developed between 750 to 1000 MHz as judged by the greater than one ratio values **D_m_/ D_air_inc_** (1.8-1.95) (Supplemental Material Fig. 5Sc). This effect we think may be attributed to an enhanced medium polarization vector value pointing parallel to the irradiating EMF when this varies between 750-1000 MHz (when the causing charged polarized species rotate synchronously with the field). However, either at the lowest tested frequency of 700 MHz or once the EMF frequency is increased (1100-1240 MHz) the measured ratio values **D_m_/ D_air_inc_** (0.4-0.5) are subunitary (Supplemental Material Fig. 5Sc). To explain this we presume at 720 MHz or above 1100 MHz the global cell medium polarization vector changes its orientation with respect to the electric vector of the irradiating EMF(at these frequencies the vector sum of polarized medium molecular species may lag the electric field vector variation with a given phase difference, opposing it and reducing its electric component effect) (Supplemental Material Fig. 5Sc). Such changes in polarization with frequency of EMF exposed liquids have been described before either for very heterogenous media or at distinct liquid-liquid electrical interfaces^11,12^.

**3S.***Methods-Irradiating EMF characterization*; *Electric flux density (displacement D), Electric field intensity (E) and Irradiative* *power flux density (S), measurements.* Two commercially available (AARONIA Near-Field 0-6 GHz) probe circular logarithmic antennas were calibrated prior to our measurements. For the investigated frequency range (720 MHz - 1224 MHz) calibration, we the same sinusoidal frequency generator (described above) with the standard Yagi emission irradiation antenna and positioned our probe antenna at 2 m from the emitter in the hood of a calibrated receiver (700 MHz - 18 GHz) horn antenna. A correspondence mapping between the two received signals (one in the probe and the other in the horn receiver antenna) was recorded and further used as a calibration data-sheet for the probe antenna recorded signals. Using the two identical probe antennas (calibrated as described) one for emission and one receiver we first estimated from measurements where inside the incubator, the EMF electric intensity varies the least. First measurements were done with the generator connected to the calibrated emission probe antenna positioned and attached adjacent to a plate well (Supplemental Material Fig. 5Sa), whereas the other receiver probe antenna was positioned inside the same well with or without RPMI cell medium with 10% FBS. The signals from the receiver probe antenna were measured and recorded at the oscilloscope (KEYSIGHT, DSOX6004A Infinii Vision 6000 X - Series with Digital Storage, 6 GHz, 20 GS/S, 4 Channels, 50 ohms resistive impedance). The plate with both probe antennas was moved inside the incubator. The plate position in the incubator found with the least deviations of EMF electric intensity values among the six wells was chosen as reference for irradiating/plate positioning (see Supplemental Material Fig 1Sa). To characterize the irradiated EMF in the incubator we used as receiver the calibrated probe antenna, placed in absence of liquid media in the plate container at distances corresponding to the sample positions x_0_, x_1_, x_2_ (see Supplemental Material Fig. 1Sb inset), and in all three orientations ($\vec{x},\vec{y},\vec{z}$) in each corresponding well^13^. For these measurements the emission broadband, 800 MHz - 3 GHz LTE ATK-LOG ALP logarithmic antenna was coupled to the generator and was held in the exact same reference position as it was during the cellular irradiation experiments (see Supplemental Material Fig 1Sa). The received voltage and current intensity signals from the receiver probe antenna connected to the same oscilloscope (as described above) were measured and recorded. The surface of the irradiating antenna elements was assessed from its constructive specifications. The recorded oscilloscope signals were used to calculate and map a topological ‘approximation’ of the power flux density S, ^13^ at the 6 points of interest in the plate wells. It is important to state that for each emission frequency, considerable local variation (within cm range distance) was recorded due to the relative position of the wells with respect to the lobes of the emitting antenna (‘near field’ zone) and due to the reflections inside the incubator ^10^. For Fig. 4 (main text) the reported values we used the ‘averaged’ values obtained for each frequency from four independent readings centered around the centers of the plate wells (see Supplemental Material Fig 1Sb). Despite our sustained efforts measuring EMF power flux density values in the incubator with the culture RPMI media inside the plate wells (in liquid), yielded relatively divergent recorded readings in very proximal positions(cm- range) due to the mentioned local effects, and for this reason here we report only in the air with CO_2_ 5 vol. %, and 95% water humidity (‘near field’ zone) incubator values. To express the effect the medium RPMI 1640 with 10% FBS has on the EMF electric intensity, we performed a new set of measurements for polarization **P** and electric flux density (displacement) field **D** vectors in the absence or presence of the liquid cell growing medium. The measurements were performed just in one direction (‘z’) assuming that the liquid has an isotropic behavior in the field and permittivity is a ‘scalar’ value^13^. The same previously calibrated probe receiver antenna was connected to an oscilloscope and placed into the central plate well whereas the other calibrated emission probe antenna branched to the EMF generator (same as the one used during cell irradiation experiments) was placed in close proximity outside the bottom wall of the polystyrene plate (Supplemental Material Fig. 5Sa and b). Voltage and impedance measured values were recorded and used to calculate in the incubator air (humidified CO2 vol. 5 % and 955 water saturated) the electric flux field density (displacement) **D_air_inc_**. Next we performed the same set of measurements with the same setup (Supplemental Material Fig. 5Sa and b), but with the central well filled with cell culture medium RPMI 1640 with 10%FBS. Similarly, the electric flux density (or displacement field) **D_m_** in the liquid medium was calculated and the ratio **D_m_**/ **D_air_inc_** values for each irradiating frequency were plotted against frequency values in Supplemental Material Fig. 5Sc, for two power dial positions of the EMF generator (either 7dBm or 13 dBm).

**SUPPLEMENTARY FIGURES**

**
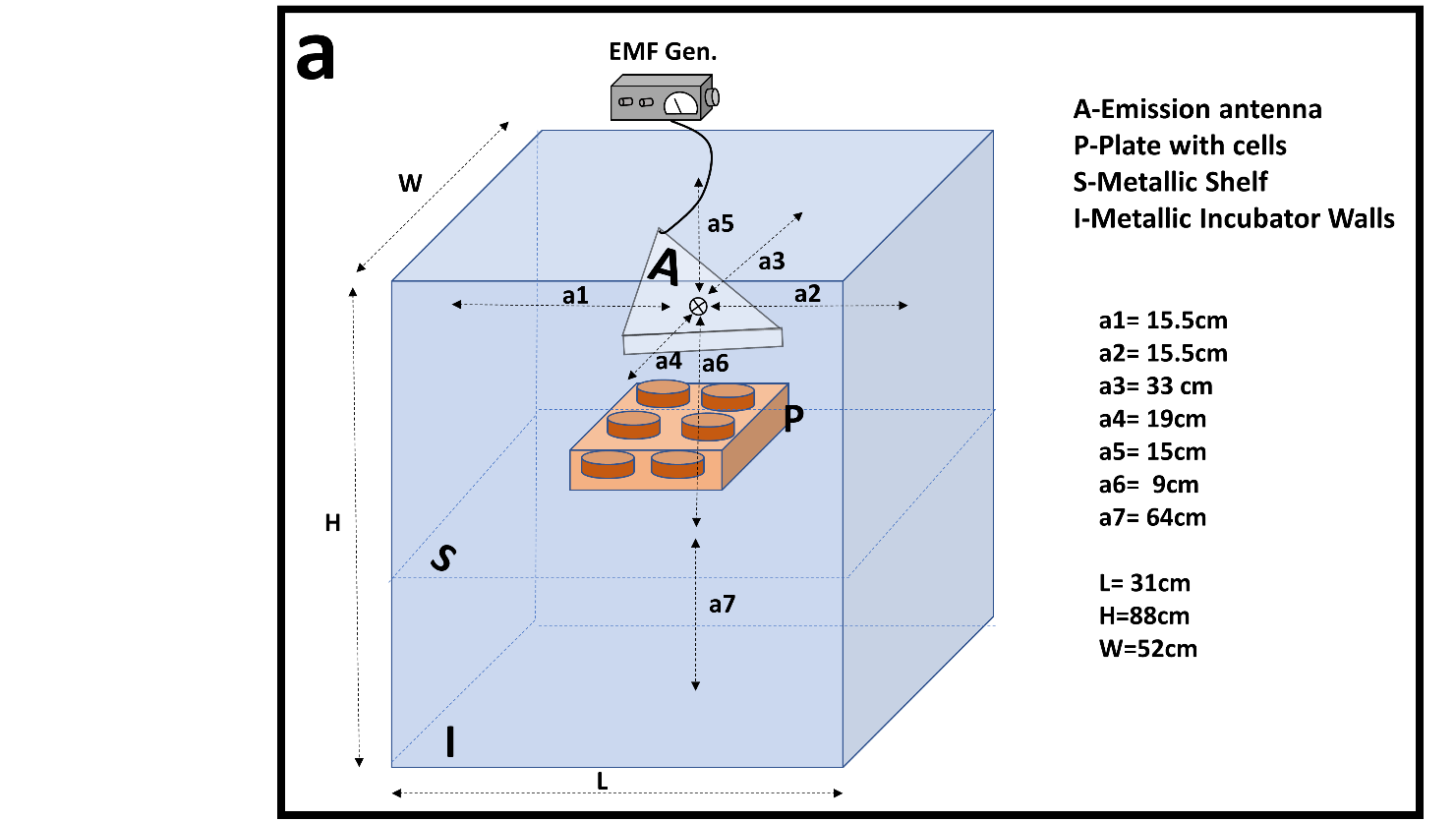
FIGURE 1S.**

**
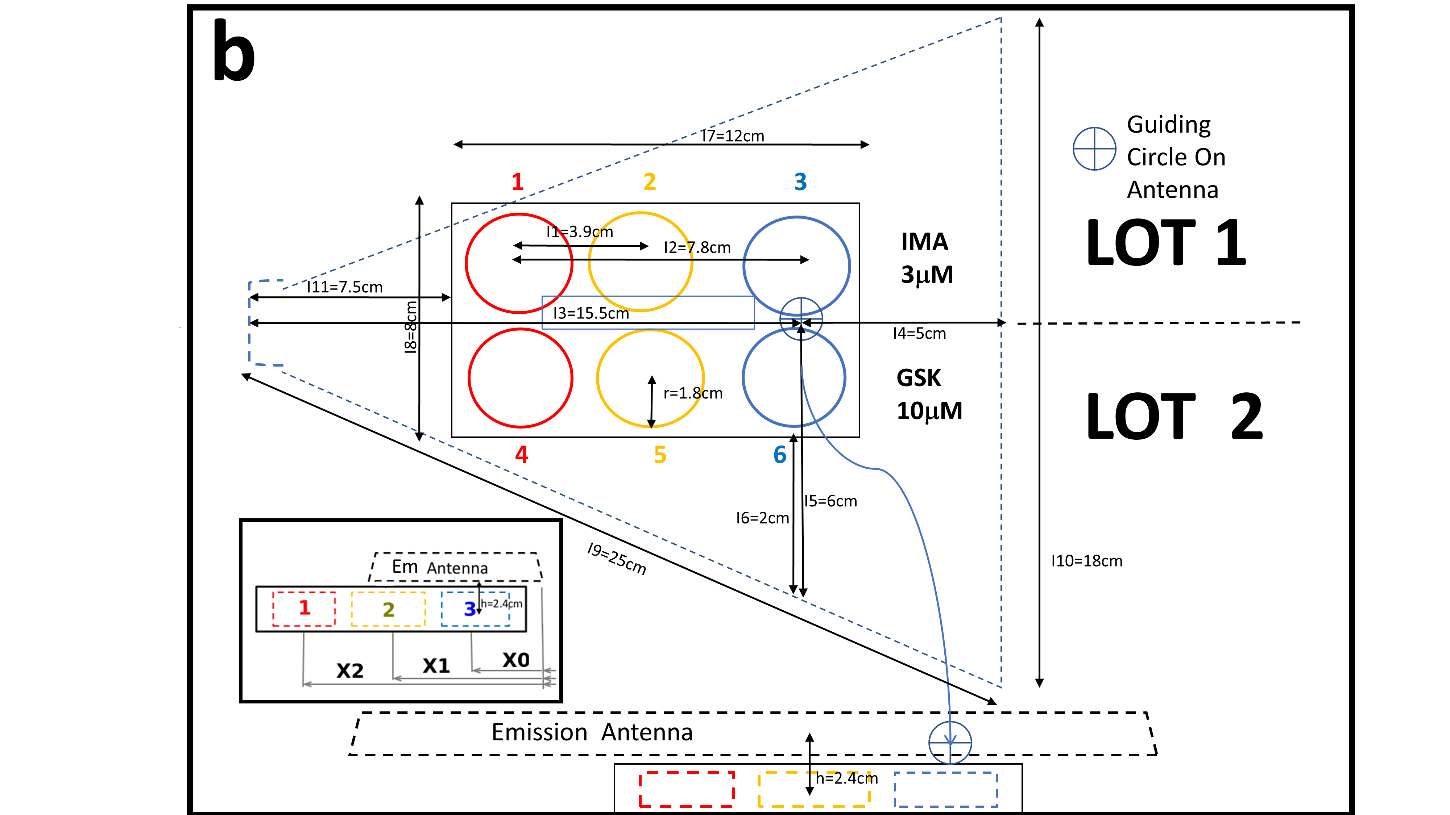
**

**Fig.1S.** The pre-B cells EMF irradiation setup. **a.** Schematic diagram depicting the spatial positioning of the emission antenna with respect to the growing cell plate and incubator walls, in the reference position. **b**. Standardized growing conditions were used with a 6 well tissue culture plate with respect to which the emission antenna was always positioned as shown in the target cross mid-way in between wells 3 and 6. Wells 1 and 4 (red), 2 and 5 (yellow) and 3and 6 (blue) are pair-wise equivalent in terms of exposure levels and were used for in parallel growing of two recombination pharmacological stimuli (Mesylate Imatinib, IMA) versus (GSK-690693, GSK). The inset displays side view denoting x_0_, x_1_ and x_2_ center wells reference points where field measurements average values were reported, and where the receiver probe antenna was positioned during measurements.

**FIGURE 2S.**

**
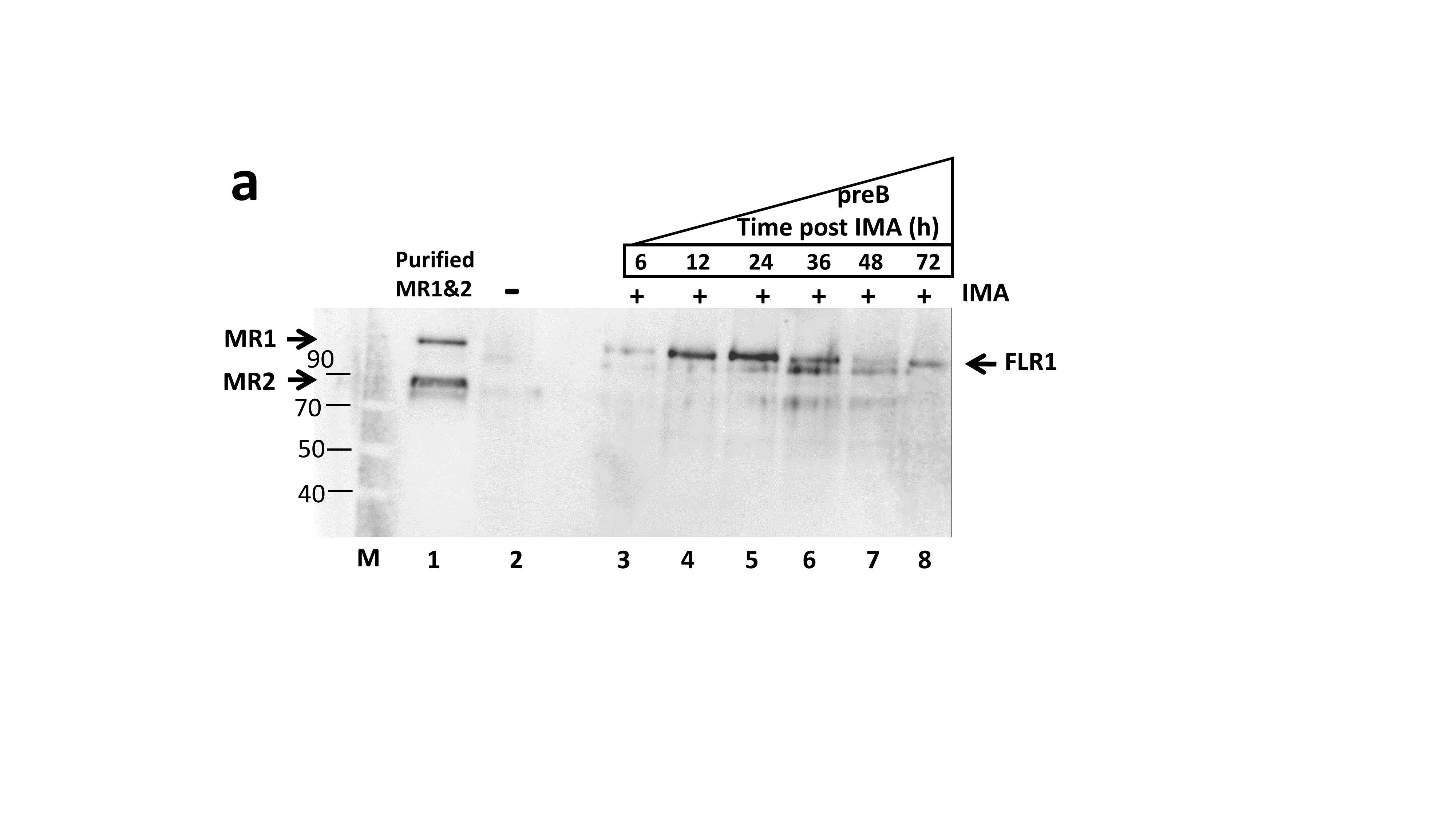
**

**
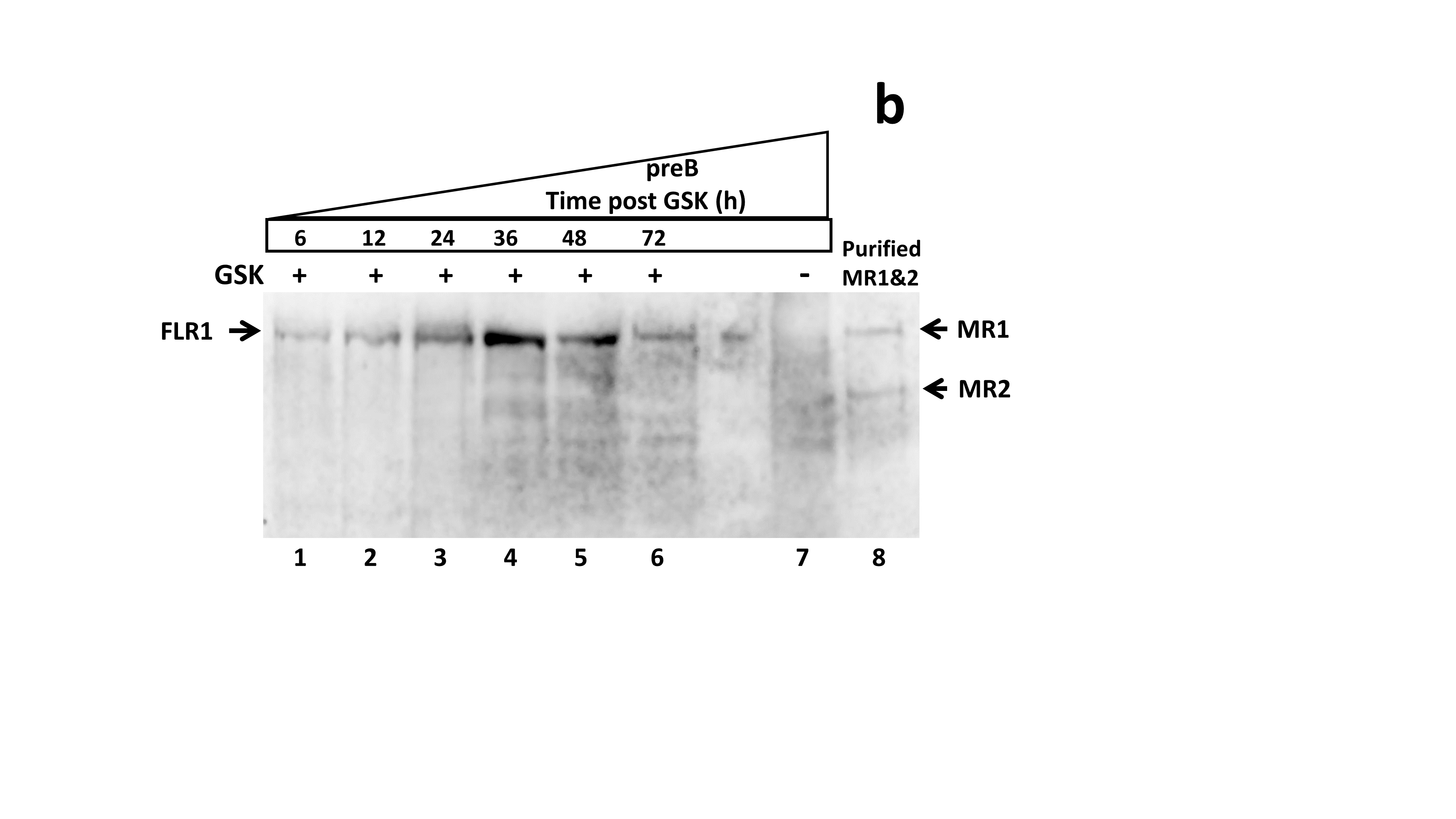
**

**Fig. 2S.** Time-course Western blot analysis of RAG1 expression in pre-B cells upon RAG induction with chemical reagents. **a.** Imatinib-IMA lanes1 to 8 denote: 1, amylose purified core RAG1(384-1040) and core RAG2(1-387) recombinant proteins fused with MBP(maltose binding protein 40kDA, MR1 and MR2), from transiently co-transfected human 293-T HEK cells**(**source ATCC^®^ CRL-3216^™^)^14^(distinct exposure), 2 v-Abl pre-B cell extract in absence of inducer(just DMSO solvent), 3 to 8 v-Abl pre-B cell extracts after 6h, 12h, 24h, 36h, 48h and 72h post 3 μM Imatinib-IMA inducer treatment. **b.** (GSK-690693, GSK) RAG1expression effects induced by GSK. Lanes 1-6. v-Abl pre-B cell extracts after 6h, 12h, 24h, 36h, 48h and 72h post 10 μM GSK-690693 inducer treatment, lane 7 no inducer. Lane 8 Purified coreRAG1 and core RAG2. coreRAG1-MBP-111.6 kDa (MR1-fused with 40kDa MBP), full length endogenous RAG1FL- 118.8 kDa, coreRAG2-MBP- 85.5 kDa (MR2 fused with MBP)^14^. The images of the blots were obtained directly by a chemiluminescence detection scanner (not from film) and in their original scans the autofocusing has trimmed some of the borders. The blots were not intentionally cut by us.

**FIGURE 3S.**

**
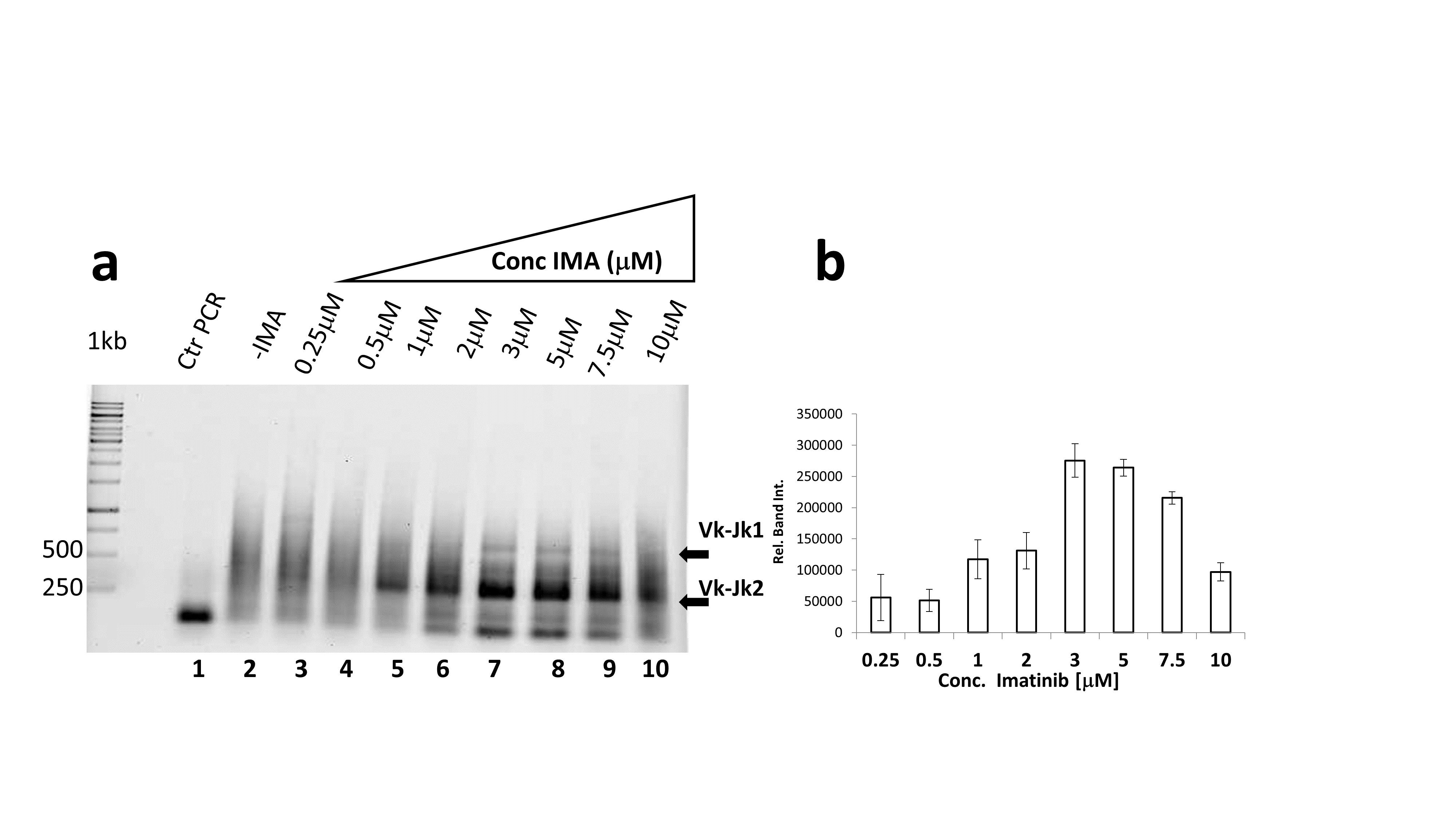
**

**
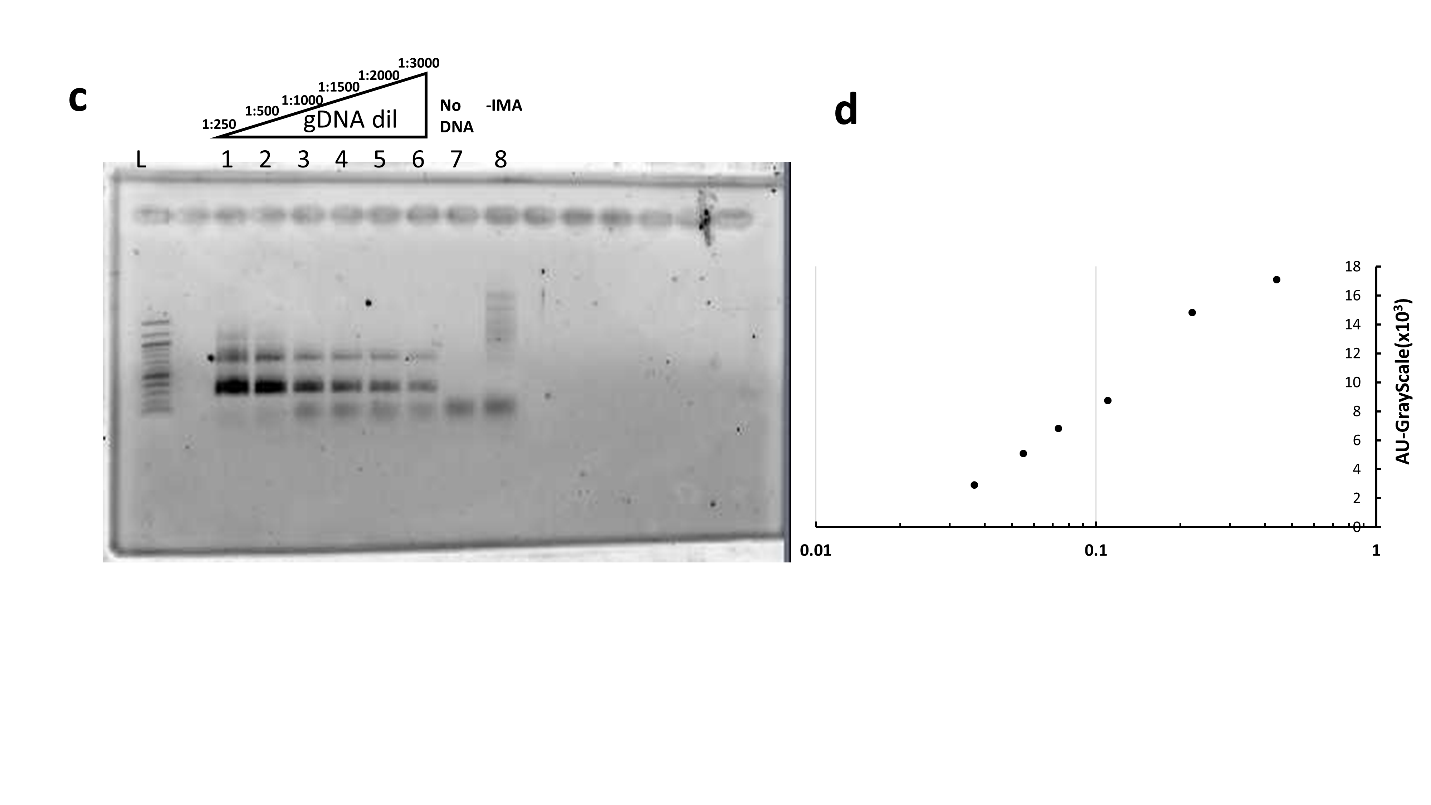
**

**Fig. 3S.** Vk to Jk recombination effects of various concentrations of IMA stimulation for 48 h of pre-B cells using the recombination PCR assay. **a.** Electrophoretic separation of amplified products lane 1 without genomic DNA, lane 2 templating genomic DNA from uninduced cells. Lanes 3-10, templating with genomic DNA extracted from 2x10^6^ cells induced with displayed IMA concentrations. **b**. Vk to Jk2 gel band densitometric quantifications expressed as histogram bars. Abscissa displays cell inducing IMA concentrations used in cell cultures, ordinate relative Band densitometric intensity of Vk-Jk2 recombination products. Shown values are the average from three independent experiments. **c**. Electrophoretic separation of amplified products; lane 7 without genomic DNA(no DNA), lane 8(-IMA) templating genomic DNA from uninduced cells. Lanes 1-6, templating with successive serial dilutions(mentioned above) of original genomic DNA extracted from 2x10^6^ cells(110ng) induced 3μM IMA. **d**. Vk to Jk2 gel band densitometric quantifications expressed as plot. Abscissa displays in logarithmic scale the exact genomic DNA dilution amount templating the first step PCR, ordinate relative Band densitometric intensity of Vk-Jk2 recombination products. Shown values are from one experiment.

**FIGURE 4S.**

**
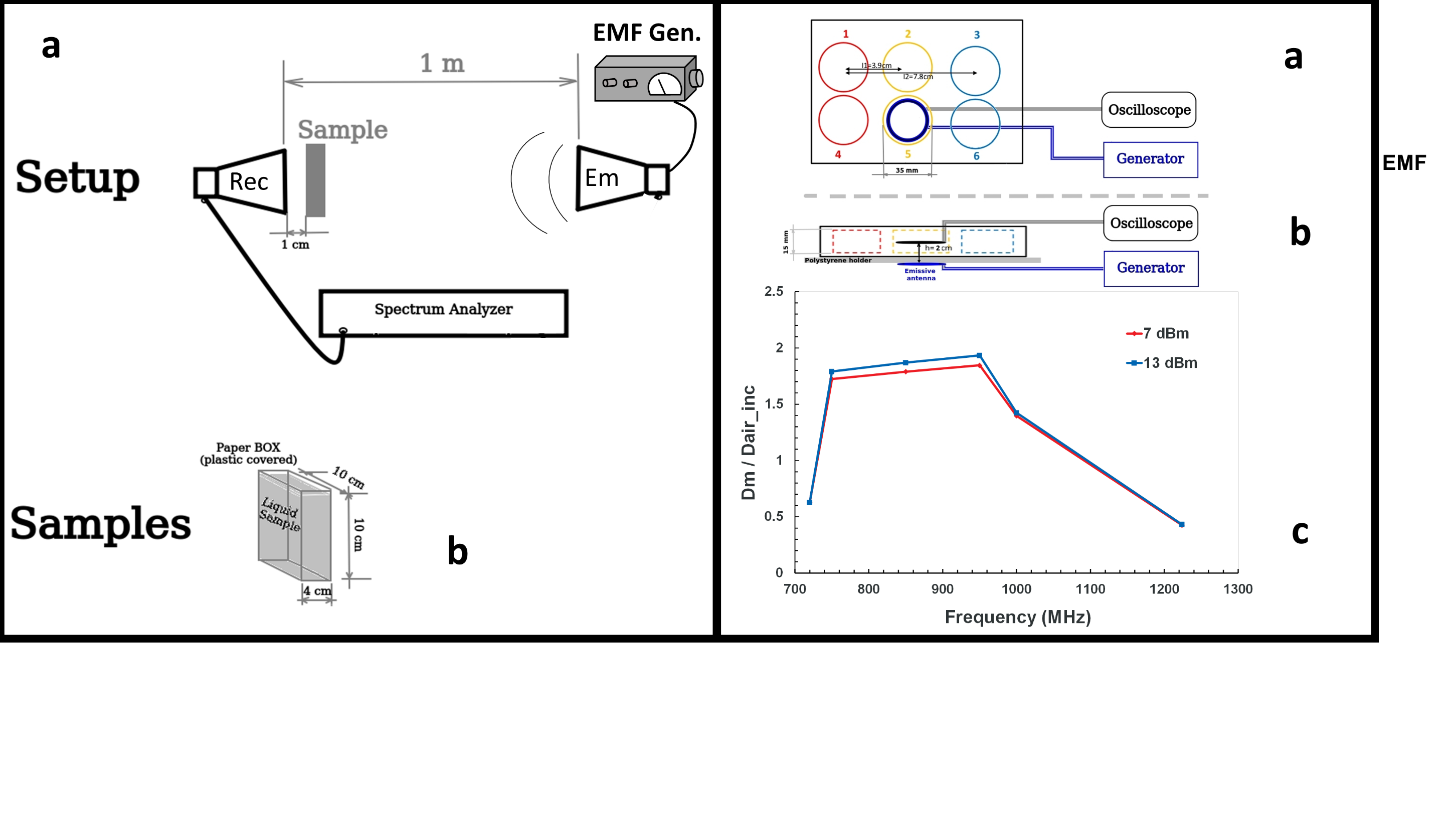
**

**Fig.4S.** Schematic Representation of the setup used for EM wave absorption spectral measurements presented in Fig.2. **a.** The spatial positioning of the sample box in 1cm vicinity of the reception antenna (Rec) and relatively distanced at 1m from the emission antenna (Em). **b.** Dimensions of the box containing the analyzed liquid samples.

**FIGURE 5S.**

**
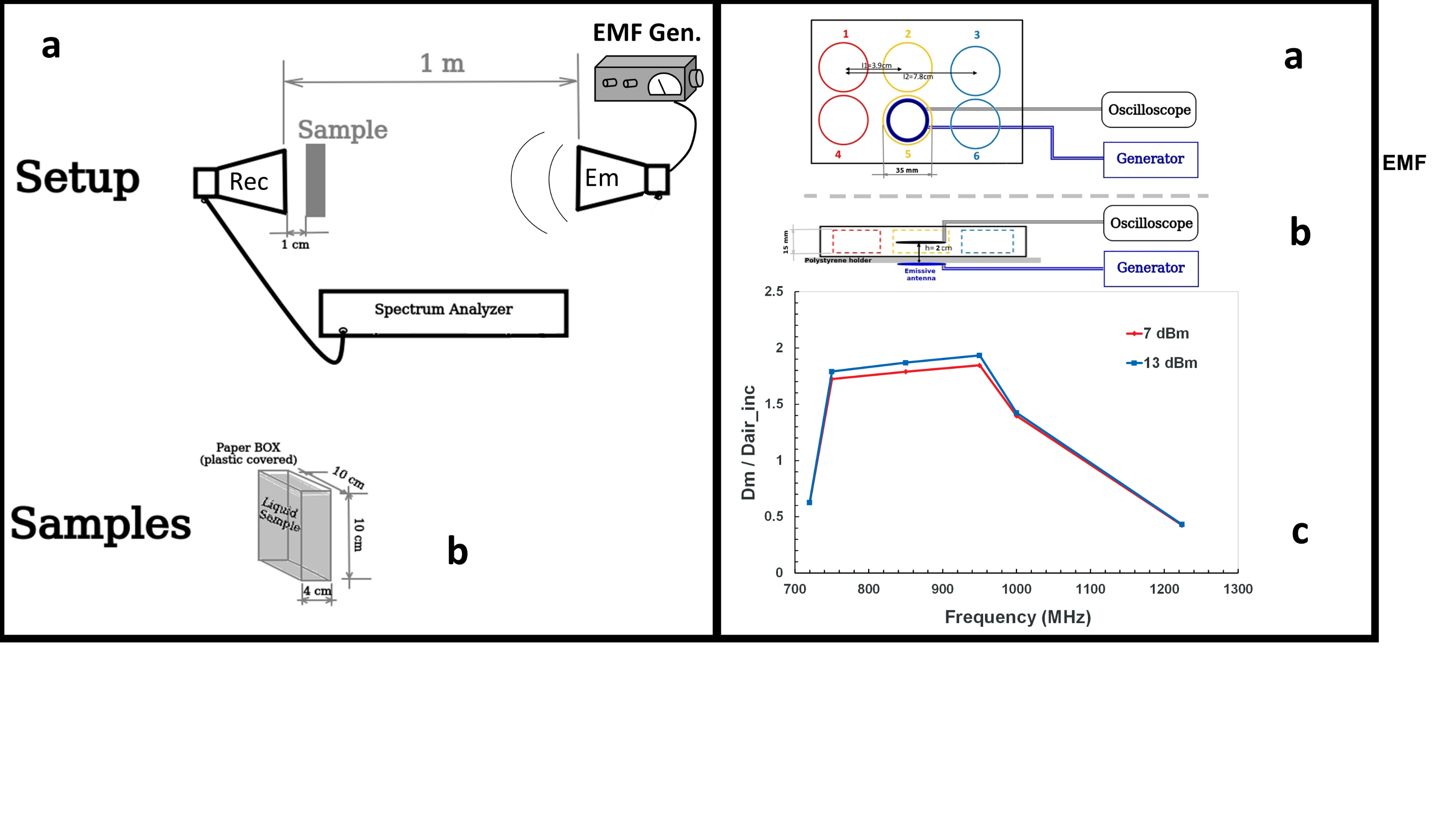
**

**Fig.5S.** Schematic Representation of EMF electric flux density measurements(D). The spatial positioning of probe receiver(gray) and of the emission(blue) antenna with respect to the plate wells. **a.** top view, **b.** side view, **c.** displays the calculated ratio of measured field electric flux density (displacement)**D_m_** values of RPMI (RPMI+10%FBS) medium versus those measured in the same place in the incubator (air_inc) **D_air_inc_,** as a function of EMF frequency at both emission power generator settings of 7 dBm (red) and 13 dBm (blue) traces. Shown values from one measurement.

**In response to the Editor request we attach the original images of scans as they were obtained from our experiments:**


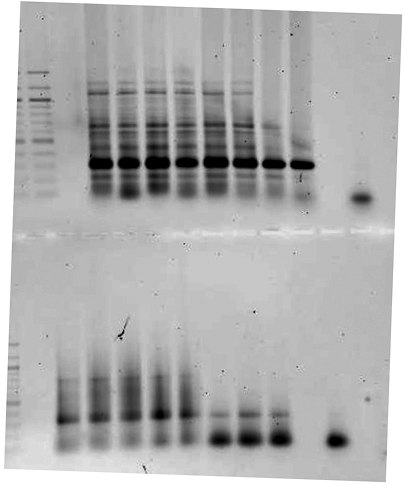


**Original fluorescence Scan of gel image shown in Fig.3A, of the main manuscript. The upper gel displays the histone H1 DNA control reactions whereas the bottom gel shows the recombination reactions.**


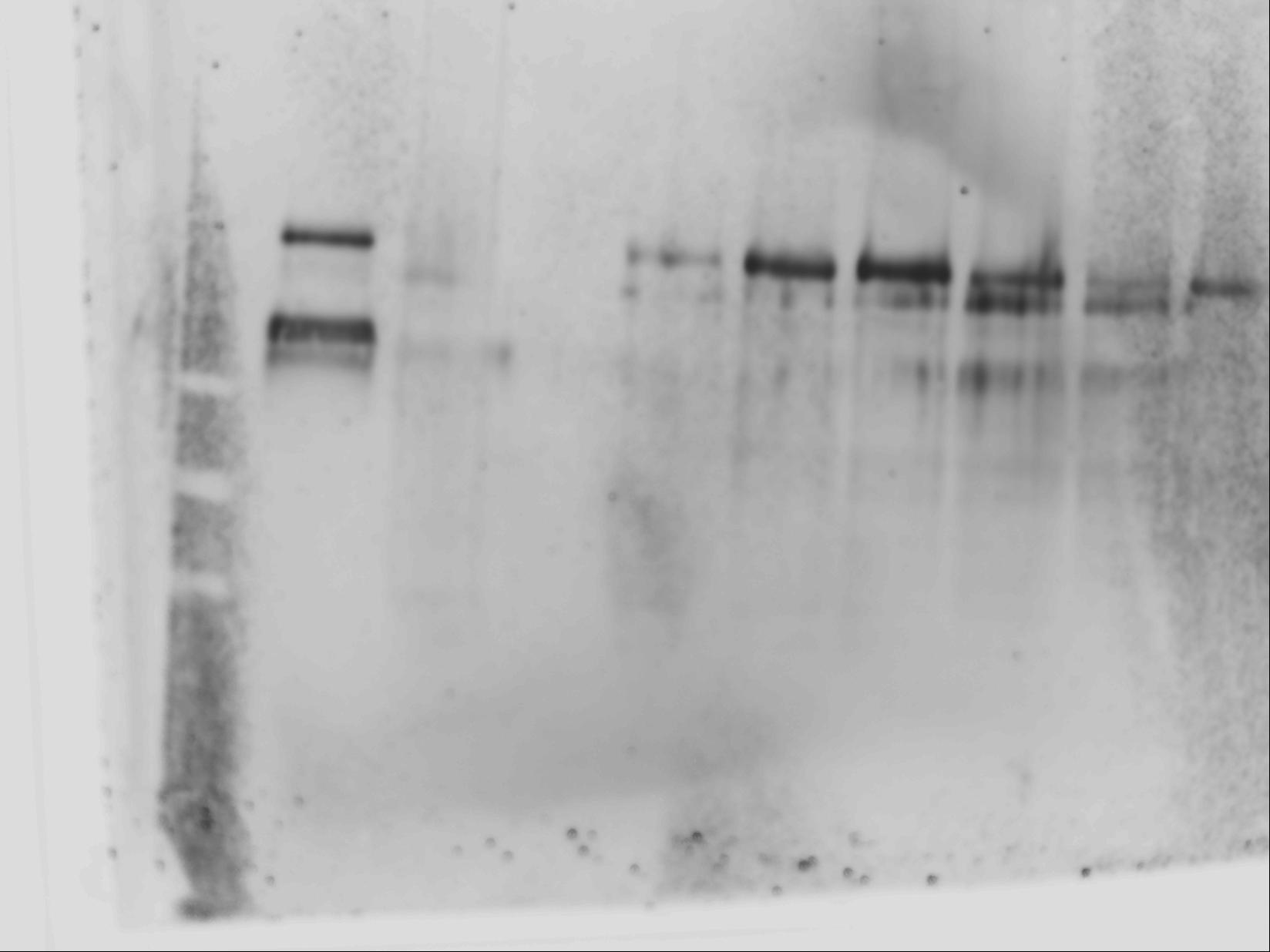


**Original blot scan described in Fig. 2Sa(in the legend of Fig.2S the boundaries of the blot borders are discussed)**


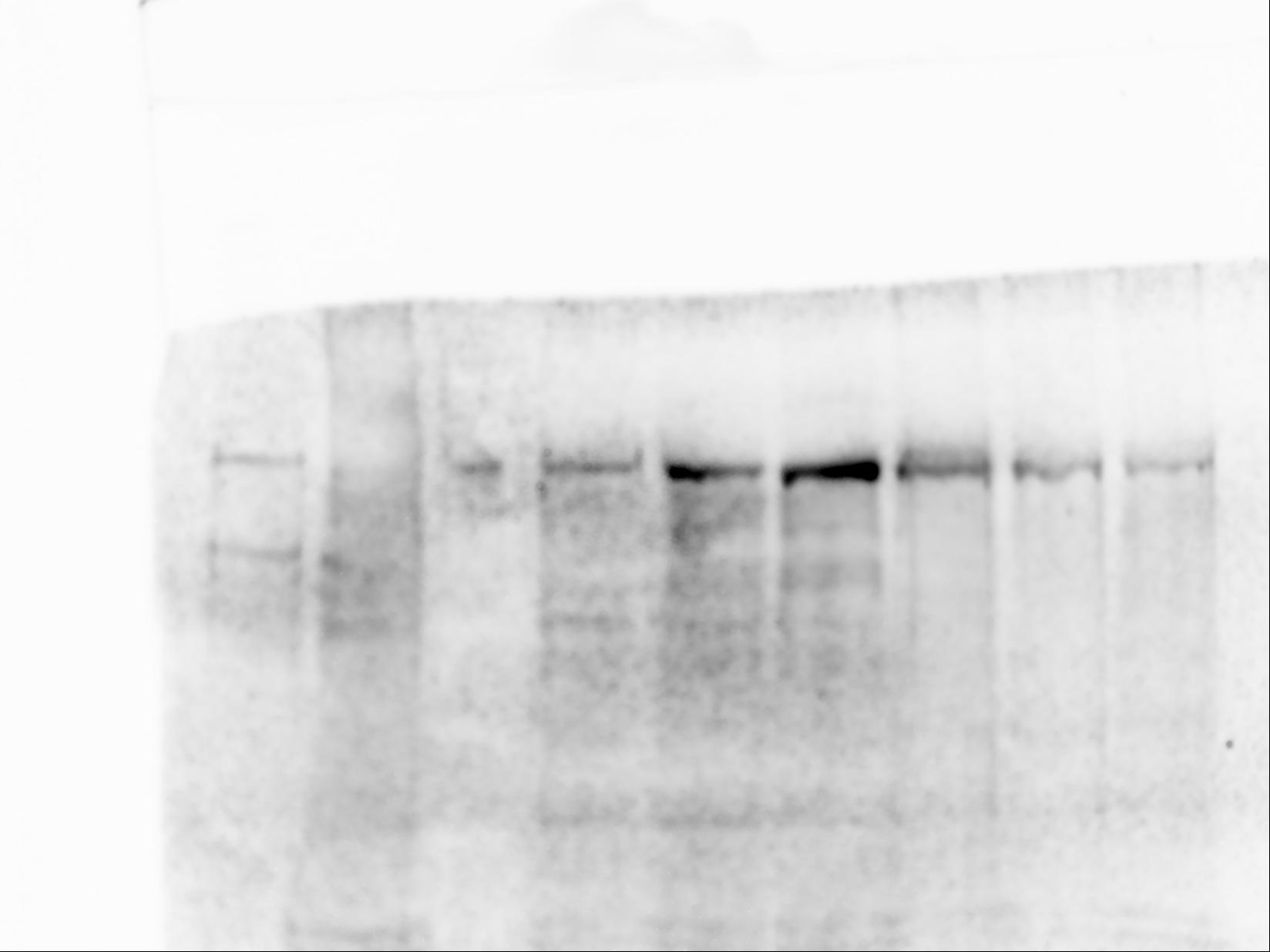


**Original blot scan described in Fig. 2Sb(in the legend of Fig.2S the boundaries of the blot borders are discussed)**

**
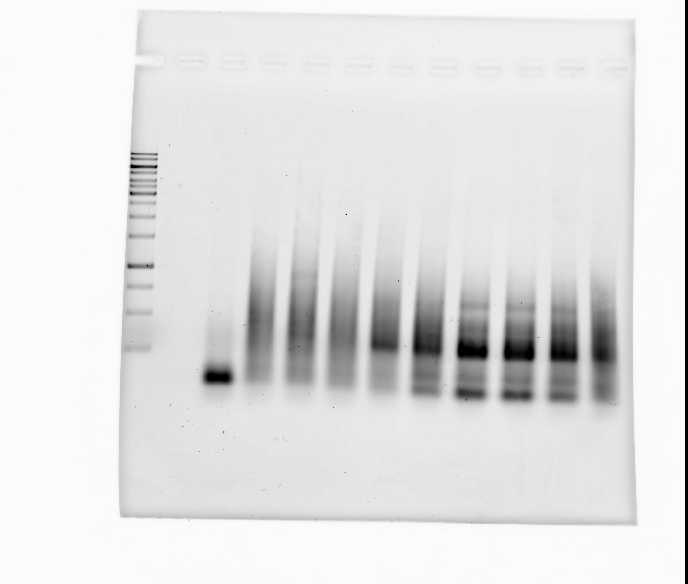
**

**Original fluorescence Scan of gel image shown in Fig.3Sa, Supplemental Material.**

**REFERENCES**

1 Muljo, S. A. & Schlissel, M. S. A small molecule Abl kinase inhibitor induces differentiation of Abelson virus-transformed pre-B cell lines. *Nature immunology* **4**, 31-37, doi:10.1038/ni870 (2003).

2 Wilson, M. K., McWhirter, S. M., Amin, R. H., Huang, D. & Schlissel, M. S. Abelson virus transformation prevents TRAIL expression by inhibiting FoxO3a and NF-kappaB. *Mol Cells* **29**, 333-341, doi:10.1007/s10059-010-0029-8 (2010).

3 Ochodnicka-Mackovicova, K. *et al.* NF-kappaB and AKT signaling prevent DNA damage in transformed pre-B cells by suppressing RAG1/2 expression and activity. *Blood* **126**, 1324-1335, doi:10.1182/blood-2015-01-621623 (2015).

4 Kuo, T. C. & Schlissel, M. S. Mechanisms controlling expression of the RAG locus during lymphocyte development. *Curr Opin Immunol* **21**, 173-178, doi:10.1016/j.coi.2009.03.008 (2009).

5 Marinelli Busilacchi, E. *et al.* Immunomodulatory Effects of Tyrosine Kinase Inhibitor In Vitro and In Vivo Study. *Biology of blood and marrow transplantation : journal of the American Society for Blood and Marrow Transplantation* **24**, 267-275, doi:10.1016/j.bbmt.2017.10.039 (2018).

6 Carmona, L. M., Fugmann, S. D. & Schatz, D. G. Collaboration of RAG2 with RAG1-like proteins during the evolution of V(D)J recombination. *Genes Dev* **30**, 909-917, doi:10.1101/gad.278432.116 (2016).

7 Borghesi, L. *et al.* B lineage-specific regulation of V(D)J recombinase activity is established in common lymphoid progenitors. *J Exp Med* **199**, 491-502, doi:10.1084/jem.20031800 (2004).

8 Constantinescu, A. & Schlissel, M. S. Changes in locus-specific V(D)J recombinase activity induced by immunoglobulin gene products during B cell development. *J Exp Med* **185**, 609-620, doi:10.1084/jem.185.4.609 (1997).

9 Grawunder, U. *et al.* Down-regulation of RAG1 and RAG2 gene expression in preB cells after functional immunoglobulin heavy chain rearrangement. *Immunity* **3**, 601-608, doi:10.1016/1074-7613(95)90131-0 (1995).

10 Portelli, L. A., Schomay, T. E. & Barnes, F. S. Inhomogeneous background magnetic field in biological incubators is a potential confounder for experimental variability and reproducibility. *Bioelectromagnetics* **34**, 337-348, doi:10.1002/bem.21787 (2013).

11 Desmond, M., Mavrogiannis, N. & Gagnon, Z. Maxwell-Wagner polarization and frequency-dependent injection at aqueous electrical interfaces. *Phys Rev Lett* **109**, 187602, doi:10.1103/PhysRevLett.109.187602 (2012).

12 Panagopoulos, D. J., Johansson, O. & Carlo, G. L. Polarization: A Key Difference between Man-made and Natural Electromagnetic Fields, in regard to Biological Activity. *Sci Rep* **5**, 14914, doi:10.1038/srep14914 (2015).

13 Gregson, S. M., J. ; Parini, C. *Principles of Planar Near-Field Antenna Measurements*. 35-61 (The Institution of Engineering and Technology, London, United Kingdom, 2007).

14 Ciubotaru, M. *et al.* The architecture of the 12RSS in V(D)J recombination signal and synaptic complexes. *Nucleic Acids Res* **43**, 917-931, doi:gku1348 [pii]10.1093/nar/gku1348 (2015).
